# Lower extremity joint-level responses to pelvis perturbation during human walking

**DOI:** 10.1038/s41598-018-32839-8

**Published:** 2018-10-02

**Authors:** Mark Vlutters, Edwin H. F. van Asseldonk, Herman van der Kooij

**Affiliations:** Department of Biomechanical Engineering, University of Twente, Enschede, The Netherlands

## Abstract

The human leg joints play a major role in balance control during walking. They facilitate leg swing, and modulate the ground (re)action forces to prevent a fall. The aim of this study is to provide and explore data on perturbed human walking to gain a better understanding of balance recovery during walking through joint-level control. Healthy walking subjects randomly received anteroposterior and mediolateral pelvis perturbations at the instance of toe-off. The open-source modeling tool OpenSim was used to perform inverse kinematics and inverse dynamics analysis. We found hip joint involvement in accelerating and then halting leg swing, suggesting active preparation for foot placement. Additionally, responses in the stance leg’s ankle and hip joints contribute to balance recovery by decreasing the body’s velocity in the perturbation direction. Modulation also occurs in the plane perpendicular to the perturbation direction, to safeguard balance in both planes. Finally, the recorded muscle activity suggests both spinal and supra-spinal mediated contributions to balance recovery, scaling with perturbation magnitude and direction. The presented data provide a unique and multi-joint insight in the complexity of both frontal and sagittal plane balance control during human walking in terms of joint angles, moments, and power, as well as muscle EMG responses.

## Introduction

Reacting to unexpected disturbances during walking is often a necessity to continue the gait cycle. A perturbation might lead to a variety of balance responses. This can depend on factors such as the magnitude and direction of the perturbation^1,2^, the point of application of the perturbation to the body^3^, the instance during the gait cycle at which the perturbation occurs^4–6^, and the objective the subject is trying to achieve^7,8^. Often, foot placement adjustments are considered as a crucial strategy to recover balance during walking^9,10^. Adequate foot placement, however, does not guarantee balance; if the leg joints do not provide the appropriate moments, the body might move in an undesired direction, or the leg might collapse with a fall as a result. Indeed, faulty weight bearing was shown to be a major cause of falls in elderly residing in long-term care^11^. Providing balance support in people prone to falls, for example through a powered lower-extremity exoskeleton, therefore requires an understanding of which joints are involved in balance recovery strategies, and what the output of these joints is.

In the field of humanoid robotics, too, there is an increasing demand for an understanding of human balance and perturbation recovery, as well as data to compare robot controller output to human balancing behavior. Data collected in humans can be used to synthesize robot motions, for example through inverse optimal control^12,13^, in which an underlying optimality criterion for a walking motion is identified based on collected data. This criterion can subsequently be used to generate movements in new walking conditions. Alternatively, neuromuscular walking models can be used to synthesize gait based on neural feedback loops from the muscles driving the leg joints^14,15^. Optimizing such models to mimic data collected in humans recovering from perturbations could provide insight in the underlying muscular and neural parameters involved in reactive balance, and help drive powered lower extremity assistive devices.

To gain a better understanding of balance during walking, several studies have focused on adjustments in step location and step duration following perturbations. Experimental results have suggested that lateral stepping modulates linearly with the mediolateral (ML) center of mass (COM) velocity, following ML pelvis perturbations^1,2^. To realize such steps the hip angles must modulate accordingly. It has also been shown that hip abductor muscle activity in the swing leg correlated with the ML distance between the COM and the stance foot during unperturbed walking^16^. These findings suggests some form of proportional swing leg control, but it is unclear whether the swing leg is simply accelerated stronger in the direction of the fall with increasing perturbation magnitude, or if the foot is actively positioned prior to foot contact, in response to a perturbation. Instruction on foot positioning can be provided through targets or stepping stones to make subjects explicitly control the location of the foot^17,18^. However, such control does not have to apply when reacting to unexpected perturbations without a distinct foot placement objective. For example, consider a rightward perturbation during left stance. In response, right hip abduction would be expected to place the right foot more rightward. Subsequently, right hip abduction would also be required after right foot contact to push the body back leftward, in the opposite direction. Hence, there might be no need to first halt leg swing prior to foot contact for positioning the foot. Finally, we previously found that wider steps as a result of ML perturbations (i.e. increased ML foot-foot distance) were generally steps of shorter length (i.e. decreased foot-foot anteroposterior distance), possibly as a consequence of a finite leg length^2^. This indicates that disturbances in one plane can also influence control in another plane. One might therefore expect increasing hip abduction to be accompanied by reduced hip flexion.

Strategies other than foot placement adjustments are also involved in stable walking^9,19^. The importance of stance-leg ankle responses in maintaining balance after a trip has been shown to prevent the body from gaining excessive angular momentum^20^. Furthermore, we previously argued that ankle moment modulation in the stance leg might assist in counteracting AP perturbations, leading to reduced need for changes in foot placement^2^. In the latter study, we found that applying anteroposterior (AP) perturbations at toe-off did not lead to significant changes in the position of the leading foot relative to the COM, at the subsequent heel strike. After foot contact, however, the center of pressure (COP) location did modulate with the perturbations, possibly indicating active ankle involvement. We have also shown the reverse; blocking the ankle joint to prevent COP modulation through ankle moments does elicit foot placement adjustments^21^. Consequently, AP perturbations are expected to result in modulation of ankle plantar- and dorsiflexor moments when such motions are possible.

The purpose of this study is to provide and explore data on perturbed human walking, to gain a better understanding of how individual joints contribute to the recovery of balance during walking. We do this for both ML and AP pelvis perturbations. The pelvis was used as point of application of the perturbation force, as it approximately coincides with the location of the whole-body COM. This prevents the perturbation from causing major body rotation, and it has allowed us to investigate the effects of changes in linear COM motion on balance responses in a controlled manner. The perturbation onset instance was at toe-off, providing subjects with the maximum amount of time for foot placement adjustments without giving them the opportunity to modulate the push-off in reaction to the disturbances. Specifically, for these perturbations we investigate (1) how the joints of the stance leg are involved in restoring the walking cycle, (2) how leg swing is regulated following perturbations and whether the swing leg is actively positioned for foot contact, (3) to what extent joint modulation occurs in the plane perpendicular to the perturbation direction. We use inverse kinematics and inverse dynamics analysis to obtain joint angles, moments, and power of the ankle, knee, and hip joints, and provide muscle activity responses of various muscles acting on those joints. We explore how these outcome variables modulate with the applied perturbations. The presented results provide insight in human balance control during walking. The joint-level output might provide guidelines for the design and control of devices to support balance in a human-like manner, for example to derive lower bounds on actuator output. The data could furthermore be used for comparison with- and validation of models and theories that attempt to explain human-like balance behavior during walking.

## Results

We introduce the following references to events in the gait cycle: toe-off right (TOR), heel strike right (HSR), toe-off left (TOL) and heel strike left (HSL). Furthermore, in the figures we refer to the end of the 150 ms perturbation window as P.end. Perturbation onset coincided with TOR. The perturbation direction could be inward (leftward for right swing leg), outward (rightward for right swing leg), forward, or backward. We highlight specific subject-average joint-level and EMG responses, resulting from transient pelvis perturbations applied randomly at the instance of TOR during slow walking. Statistical analysis was only used to substantiate statements regarding the highlighted data, indicated by a “×” in the text and the corresponding figures. Statistical analysis was not used to analyze all data, given its large amount, nor was a high-level descriptor derived from the data for statistical testing, as this would no longer reflect a joint level. We present slow walking data as these sometimes show more pronounced effects as compared to data from the normal walking speed, for example in the knee moments. This is possibly because the perturbations cause larger shifts in movement velocity relative to the intended walking speed. The complete set of results for both slow and normal walking speeds can be found in the Supplementary Materials.

All data is shown dimensionless, with joint angles presented in radians, and with joint moment scaled according to Hof ^22^. Joint power was scaled according to Pinzone *et al*.^23^, and is presented in the Supplementary Material. Subject-average scaling factors for the presented data are 1215 ± 259 Nm (m*g*l_0_) for moments and 2739 ± 562 W (m*g^1/2^*l_0_^3/2^) for power, where m is the subject mass, g is the Earth’s gravitational acceleration, and l_0_ is the subject height. Finally, the joint moments and power are not shown for inward perturbations, as these often lead to cross-stepping in which subjects stood with both feet on the same force plate during the recovery. This yields incomplete data for the inverse dynamics.

### Direct mechanical effects

**Figure 1:**
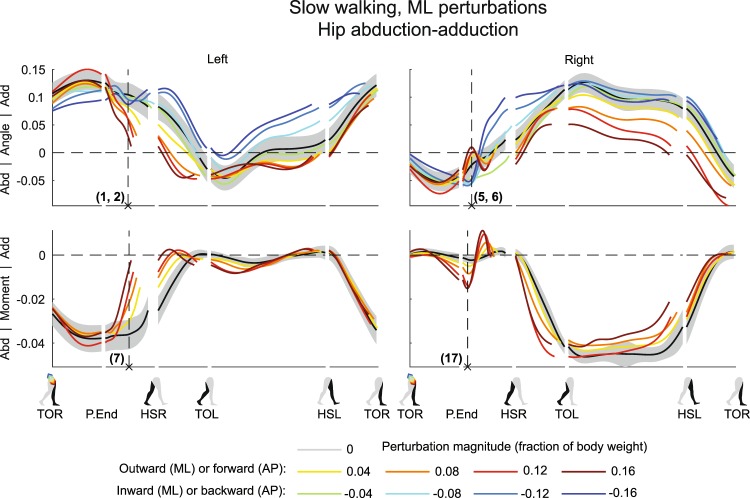
Subject-average angles, moments, and power for left and right hip ab/adduction after ML perturbations. Data is split into sequences, and shown for one gait cycle after perturbation onset at TOR (toe-off right). Subsequent events are P.End (perturbation end), HSR (heel-strike right), TOL (toe-off left), HSL (heel-strike left), and again TOR. The horizontal length of each line in a sequence reflects the duration of that sequence relative to the other conditions. Angles are in radians. Moment and power are dimensionless, with subject average scaling factors of 1215 ± 259 Nm and 2739 ± 562 W, respectively. Colors indicate the perturbation magnitudes. Yellow-red corresponds with outward perturbations, green-blue with inward perturbations. The gray shading is the subject-average standard deviation, only shown for the unperturbed condition to prevent image cluttering. In case of inward perturbations, joint moments and power are left out due to cross-stepping with both feet on the same force plate. Abd: abduction. Add: adduction.

**Figure 2:**
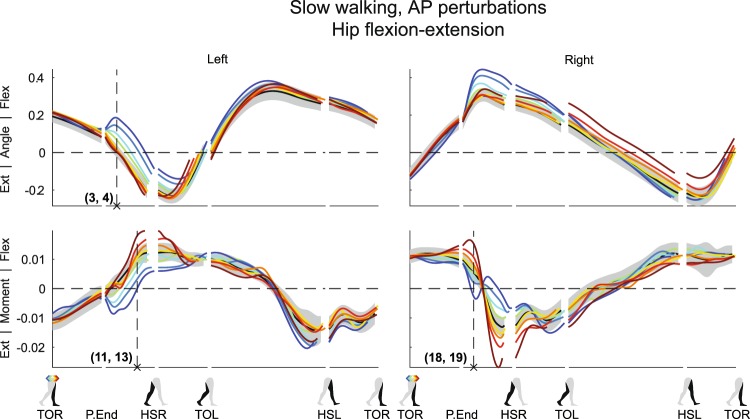
Subject-average angles, moments, and power for left and right hip flexion/extension after AP perturbations. Yellow-red corresponds with forward perturbations, green-blue with backward perturbations. Ext: extension. Flex: flexion. Please also refer to the caption and legend of Fig. 1.

The left leg was the stance leg during the perturbations. As the foot remained stationary on the treadmill belt and the body moved in the direction of the perturbations, direct mechanical effects of the perturbations are expected to occur in the stance leg joints. These effects were best reflected in the hip angles of the stance leg after the perturbations, and prior to HSR, see Figs 1 and 2.

As expected from a pure mechanical point of view, completion of outward perturbations made the left hip move more toward abduction (Fig. 1) (×1, F_4,32_ = 12.708, p < 0.001). Inward perturbations did not lead to a significant deviation in left hip abduction angle at the same time instance after perturbation onset (×2, F_4,32_ = 0.257, p = 0.903), though this angle tended more towards adduction in subsequent gait phases. Forward perturbations resulted in little deviation in left hip flexion-extension angle compared to the unperturbed condition (Fig. 2) (×3, F_4,32_ = 0.692, p = 0.603), possibly because the perturbation had the same direction as the desired movement. Backward perturbations, however, resulted in increased left hip flexion (×4, F_4,32_ = 33.485, p < 0.001), which can be partially attributed to pelvis tilt (see Supplementary Figures). In addition, the right swing leg might also experience direct effects of the perturbations. For example, directly after completion of the ML perturbations, the right hip joint showed minor adduction angles following outward perturbations (×5, F_4,32_ = 14.420, p < 0.001), and tended more toward abduction following inward perturbations (×6, F_4,32_ = 2.321, p = 0.078). This was likely caused by the body dynamics and swing leg inertia. Since this leg movement was opposite of what is required for foot placement in the direction of the fall, it indicates the necessity for active swing leg control.

### Stance leg control during the ongoing step

**Figure 3:**
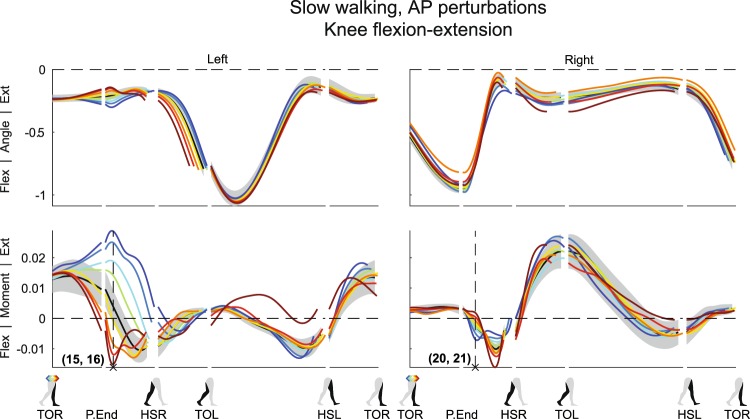
Subject-average angles, moments, and power for left and right knee flexion/extension after AP perturbations. Yellow-red corresponds with forward perturbations, green-blue with backward perturbations. Ext: extension. Flex: flexion. Please also refer to the caption and legend of Fig. 1.

**Figure 4:**
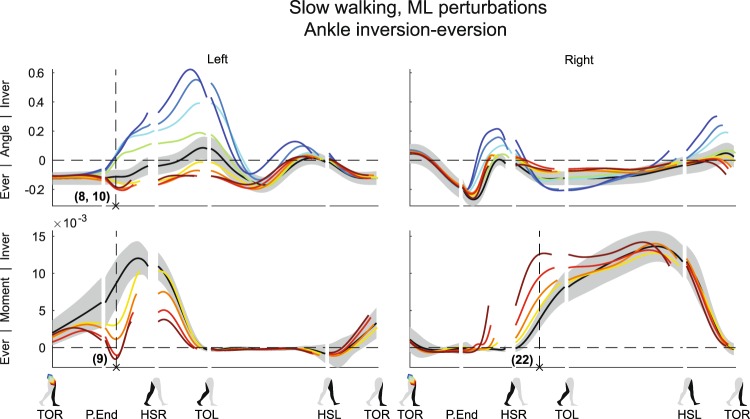
Subject-average angles, moments, and power for left and right ankle in/eversion after ML perturbations. Yellow-red corresponds with outward perturbations, green-blue with inward perturbations. Ever: eversion. Inver: inversion. Please also refer to the caption and legend of Fig. 1.

**Figure 5:**
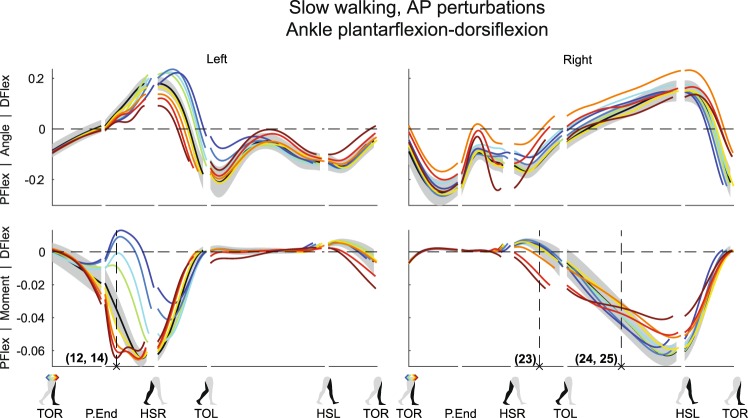
Subject-average angles, moments, and power for left and right ankle plantar/dorsiflexion after AP perturbations. Yellow-red corresponds with forward perturbations, green-blue with backward perturbations. Pflex: plantar flexion. Dflex: dorsiflexion. Please also refer to the caption and legend of Fig. 1.

The perturbations changed the velocity of the body such that, with exception of the forward perturbations, movement deviates from the desired direction. Though foot placement adjustments could help redirect the body back toward the desired movement direction, the stance leg was already used to counteract the disturbance before right heel strike occurs after a perturbation. This is reflected in the left hip joint, see Figs 1 and 2, as well as in the left knee joint, see Fig. 3, and the left ankle joint, see Figs 4 and 5, all prior to HSR.

As the outward perturbations already induced left hip abduction angles, subjects intervened by delivering less left hip abduction moment compared to the unperturbed condition (Fig. 1) (×7 F_4,32_ = 31.585, p < 0.001). However, they did not generate a left hip adduction moment in an attempt to directly oppose the perturbation-induced left hip abduction angle. Possible reasons are that not sufficient moment can be delivered to counter the sideways fall anyway, or because such moments could result in unwanted pelvis or trunk rotations. For the left ankle joint, outward perturbations yielded a minor increase in eversion angle (Fig. 4) (×8 F_4,32_ = 10.462, p < 0.001). The ankle moment also tended toward eversion (×9, F_4,32_ = 30.850, p < 0.001), especially for perturbations of larger outward magnitude. This allowed the COP beneath the left stance foot to move more medially, in the direction of the fall. In contrast, inward perturbations lead to strong left ankle inversion angles (×10, F_4,32_ = 15.033, p < 0.001), moving the COP laterally, also in the direction of the fall.

In the AP direction, the hip and ankle joints of the stance leg helped oppose the AP perturbation effects. In response to forward perturbations, subjects delivered an increased left hip flexion moment (Fig. 2) (×11, F_4,32_ = 13.384, p < 0.001). This slows down forward movement, and at the same time facilitates forward swing when the left leg transfers from stance to swing between HSR and TOL. Furthermore, these moments could have assisted in keeping the trunk upright, which will tend to flex backward after the pelvis is pushed forward. The left ankle delivered more plantar flexion moment (Fig. 5) (×12, F_4,32_ = 58.351, p < 0.001), which also slows down forward movement as long as the foot is flat on the floor and does not pitch over the toes. For backward perturbations the opposite occurs; subjects delivered more left hip extension moment (×13, F_4,32_ = 18.250, p < 0.001), to negate the perturbation-induced left hip flexion (Fig. 2). Meanwhile, the left ankle showed a strong reduced plantar flexion moment, or even a dorsiflexion moment (Fig. 5) (×14, F_4,32_ = 250.019, p < 0.001). Dorsiflexing the stance foot minimizes braking, helps the body fall forward, and allows it to pick up speed again.

Finally, subjects appeared to maintain the unperturbed left knee angle following AP perturbations (Fig. 3). They generated less extension moment, or even a flexion moment to prevent the knee from over-extending following forward perturbations (×15, F_4,32_ = 80.027, p < 0.001). Conversely, they generated more extension moment to prevent excessive knee flexion following backward perturbations (×16, F_4,32_ = 55.508, p < 0.001). As for the ankle and hip joints, deviations in the knee from the unperturbed condition increased with increasing perturbation magnitude.

### Swing leg control ensures foot positioning

A fall will likely occur if swing leg movement is not actively adjusted to change the location and/or timing of foot placement following the perturbations. Subjects changed their swing leg control in response to the perturbations, as can be seen in the right hip joint, see Figs 1 and 2, as well as in the right knee joint, see Fig. 3.

In the ML direction, following outward perturbations, subjects delivered a right hip abduction moment to swing the leg outward and realize a more lateral foot placement (Fig. 1) (×17, F_4,32_ = 25.459, p < 0.001). This moment increased in amplitude with increasing perturbation magnitude. However before HSR this abduction turned into an adduction moment, halting further right hip abduction. After HSR, the right leg becomes the new stance leg, and subjects again delivered an abduction moment. This pushes the body back in the direction from which it came, and prevents it from laterally toppling over the leg.

Similar moment reversals were observed in the AP direction. To facilitate forward leg swing, subjects delivered more right hip flexion moment with increasing forward perturbation magnitude (Fig. 2) (×18, F_4,32_ = 39.572, p < 0.001), even though right hip flexion-extension angles initially do not appear to strongly deviate from the unperturbed condition. Then, before HSR, this moment was reversed into a right hip extension moment, which halts forward leg swing. The opposite occurred for backward perturbations; subjects initially delivered less right hip flexion moment as compared to the unperturbed condition, or even generate an extension moment (×19, F_4,32_ = 10.498, p < 0.001). Subsequently, for all backward perturbations a hip extension moment was delivered prior to HSR to halt forward leg swing.

Finally, the right knee also showed such moment fluctuations as compared to the unperturbed condition (Fig. 3). Following forward perturbations first less flexion moment was delivered (×20, F_4,32_ = 24.934, p < 0.001), only to deliver more flexion moment slightly later within the same gait phase, before HSR. This might be related to movement of the upper leg, which can influence knee flexion-extension through inertial effects of the lower leg. Again, a similar but opposite response occurred for backward perturbations (×21, F_4,32_ = 7.003, p < 0.001).

### Ankle control after first heel contact

The right leg further contributed to the perturbation recovery after HSR. Here we highlight the right ankle joint, see Figs 4 and 5. Following outward perturbations, subjects delivered more right ankle inversion moment just before HSR (×22, F_4,32_ = 35.752, p < 0.001), and continuing after HSR (Fig. 4). Such moments shift the COP laterally in the direction of the perturbation, and likely help the body return toward the direction from which it came. Forward perturbations resulted in additional ankle plantar flexion moments between HSR and TOL (×23, F_4,32_ = 35.887, p < 0.001), to counteract the gained forward velocity (Fig. 5). Prior to HSL, however, less right ankle plantar flexion moment was generated as compared to the unperturbed condition, possibly to generate less push-off (×24, F_4,32_ = 0.832, p = 0.515). For backward perturbations no major changes occurred directly after HSR, but some additional right ankle plantar flexion moment is generated between TOL and HSL (×25, F_4,32_ = 1.687, p = 0.177), possibly to generate forward velocity through additional push-off. For the latter two responses (×24, 25) only the intercept of the linear mixed model tested significant (p < 0.001), suggesting all perturbations lead to a similar deviation in ankle moment, regardless of the perturbation magnitude.

### Perturbations also evoke perpendicular responses

**Figure 6:**
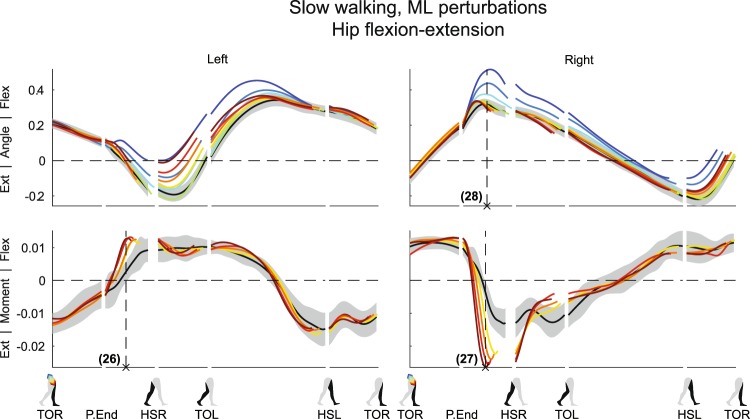
Subject-average angles, moments, and power for left and right hip flexion/extension after ML perturbations. Yellow-red corresponds with forward perturbations, green-blue with backward perturbations. Ext: extension. Flex: flexion. Please also refer to the caption and legend of Fig. 1.

The perturbations not only affected movement in the direction in which they were applied, but also evoked responses perpendicular to the perturbation direction. These responses also scaled with the perturbation magnitude. This can be seen in the hip flexion-extension responses following ML perturbations, see Fig. 6, but also in the ankle responses (Figs S1 and S9).

For the left stance leg during and directly after the ML perturbations, subjects delivered an increased left hip flexion moment following all outward perturbations (×26, F_4,32_ = 17.865, p < 0.001). This flexion moment slows down forward progression, and might be in preparation to handling the decreased AP distance to the (right) leading foot at HSR that accompanies wider steps^2^. It might help prevent toppling over the leading foot too quickly once it is placed at a shorter AP distance from the body.

As in the left leg, the right hip flexion-extension moments also modulated with outward perturbations. Subjects delivered an increased hip extension moment prior to HSR to slow forward leg swing (×27, F_4,32_ = 61.019, p < 0.001). This helps move the foot toward the floor in preparation for an earlier HSR. Finally, for inward perturbations, increases in right hip flexion angle were observed (×28, F_4,32_ = 44.741, p < 0.001), likely required to move the right leg over the left leg when making a cross-step.

### Muscle activity reveals spinal and supra-spinal mediated responses

**Figure 7:**
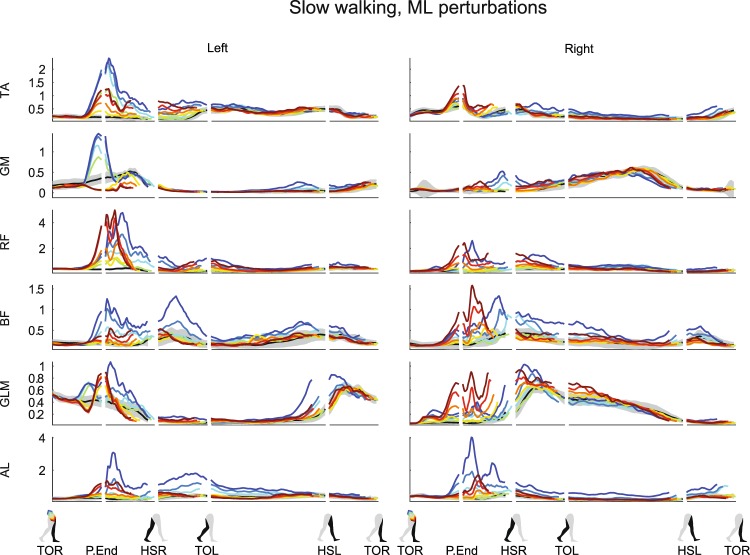
Subject-average EMG responses for various muscles of the left and right leg after ML perturbations. Yellow-red corresponds with outward perturbations, green-blue with inward perturbations. TA: tibialis anterior. GM: gastrocnemius medialis. RF: rectus femoris. BF: biceps femoris. GLM: gluteus medius. AL: adductor longus. Please also refer to the caption and legend of Fig. 1.

**Figure 8:**
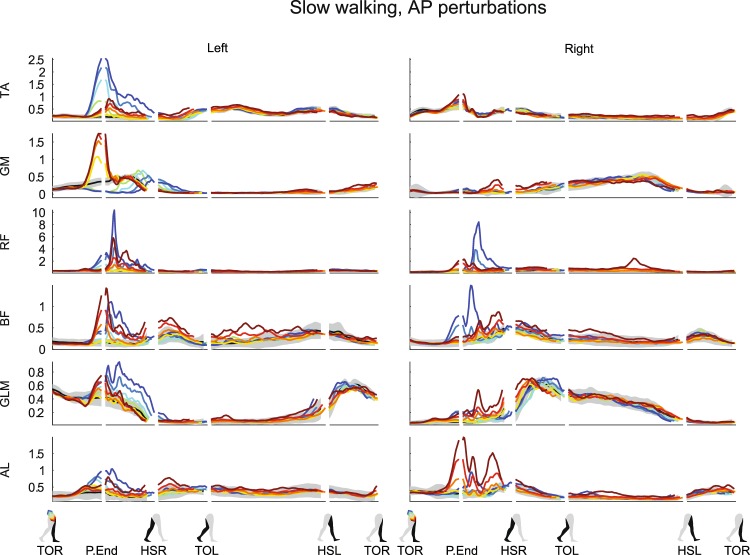
Subject-average EMG responses for various muscles of the left and right leg after AP perturbations. Yellow-red corresponds with forward perturbations, green-blue with backward perturbations. TA: tibialis anterior. GM: gastrocnemius medialis. RF: rectus femoris. BF: biceps femoris. GLM: gluteus medius. AL: adductor longus. Please also refer to the caption and legend of Fig. 1.

All recorded muscles, in both legs, visually appeared affected by both the ML and the AP perturbations. Activity modulation mainly occurred before the HSR following perturbation onset. Responses often depended on the perturbation direction, and scaled with the perturbation magnitude, see Fig. 7 for ML perturbations and Fig. 8 for AP perturbations. The earliest response onsets were located in the right gluteus medius (GLM) following ML perturbations, typically in the range of 70 ms (62 ± 19 ms for 0.16 inward, 81 ± 25 ms for 0.16 outward). Such latencies suggest a reflexive origin, most likely caused by perturbation-induced changes in muscle length and tendon force. For the left GLM, the deviation onset is typically detected as 150 ms, at P.End (148 ± 48 ms for 0.16 inward, 146 ± 26 ms for 0.16 outward), based on the deviation criterion above three times the baseline repetition standard deviation of the unperturbed EMG that was set. Other muscles with a clear onset include the left tibialis anterior (TA) for ML perturbations (113 ± 20 ms for 0.16 inward, 131 ± 10 ms for 0.16 outward), as well as AP perturbations (116 ± 9 ms for 0.16 backward), and the left gluteus medius (GLM) for inward perturbations (133 ± 32 ms for 0.16 inward) and forward perturbations (131 ± 30 ms for 0.16 forward). Even muscles which are unlikely to experience direct mechanical effects of the perturbations showed modulation with the perturbation magnitude, starting around 100–150 ms after perturbation onset. Examples include the right TA (141 ± 19 ms for 0.16 outward), rectus femoris (RF, 141 ± 36 ms for 0.16 inward, 110 ± 17 ms for 0.16 outward) and biceps femoris (BF, 148 ± 30 ms for 0.16 inward, 136 ± 27 ms for 0.16 outward) muscles following ML perturbations. Supra-spinal neural structures can be involved in these responses, given their latency^24^. Most often the activity increased, but the left GLM muscle and the left GM muscle show that activity decreases were induced as well.

Various perturbations lead to co-contractions. The TA and GM, the RF and BF, and the GLM and Adductor longus (AL) muscle pairs in the left leg all appear to show activity increases following larger inward perturbations. These could result an increased impedance in all the stance leg joints, keeping the leg stable while the body laterally topples over it. Finally, in many cases the muscle activity corresponds to the moments from the inverse dynamics, such as the strong increase in the left TA muscle and the activity drop in the left GM muscle following backward perturbations, causing the observed dorsiflexion ankle moments.

## Discussion

We aimed to explore joint-level contributions to countering unexpected pelvis perturbations during walking, for joints in both the stance leg and the swing leg. Healthy human subjects were perturbed during walking at the instance of toe-off right. Inverse kinematics and inverse dynamics analysis were used to investigate joint-level responses to the perturbations. Shortly after a perturbation the joint angles and moments, as well as the activity levels in various leg muscles, often scaled with perturbation magnitude and direction. This occurred in multiple joint and muscles simultaneously. Not simply a single joint is affected, nor is a single joint primarily responsible for rejecting the perturbation-induced effects. Instead, a multitude of actions ensures a return to the unperturbed walking cycle. This dataset reveals the complexity of the responses, and provides a unique, multi-joint insight in both the frontal and sagittal planes of balance control during walking.

Though foot placement adjustments are often considered the most important strategy to maintain balance during walking, it is complemented by various other recovery strategies. In our experiments, we have suppressed one such strategy by asking subjects to walk with the arms over the abdomen, which might affect the natural balance response. However, we expect swaying of the arms to have minor contributions compared to that of the legs. None of the subjects showed strong urges to sway the arms in response to the disturbances. The remaining recovery strategies can be addressed in various combinations, still allowing for an abundance of recovery options. This is demonstrated by the stance leg, which is addressed before the first heel strike occurs after the perturbations. For example, the left hip extension moments paired with the dorsiflexion moments in the left ankle joint allow the body’s COM to pick up forward speed following backward perturbations. Such actions reduce the need for foot placement adjustments, which alternatively could also result in successful balance recovery. Other stance leg actions could include preventing the COM from losing height, such as by timely stance leg extension through ankle plantar flexion following forward perturbations. Preventing the COM from losing height could be beneficial as redirecting the COM back upward is costly^25^. Other recovery options, available after foot contact, might also reduce the need for foot placement adjustments. Given the dimensions of the human foot, ankle joint moments delivered after foot contact are suitable for further slowing down forward movement and rejecting forward perturbations. These actions might explain why we previously found little adjustment in foot placement location relative to the COM following AP perturbations^2^.

The swing leg is actively positioned to realize foot placement adjustments, as indicated by the hip joint moments that halt the leg swing, as well as by the corresponding EMG activity. The leg is not simply swung in the direction of the fall until foot contact occurs due to the fall itself. It remains inconclusive, however, whether humans explicitly regulate the location of the foot and the timing of foot placement, or control some other variables. While it is possible to control the foot location, for example when stepping towards targets^17,18^, accurate foot placement is not a prerequisite for balance, nor does it guarantee balance. It merely provides a window of opportunity for the leading leg to redirect body motion after foot contact. Rather than explicitly controlling the location and timing of foot placement, we perhaps learn coordinated muscle activation patterns that achieve objectives related to whole-body balance, as has been suggested for balance strategies in standing^26,27^. An optimal way to balance involving foot placement adjustments might be learned through trial-and-error, involving many steps and falls^28^. Such prior experience might be addressed when faced with a new, unexpected perturbation.

The responses occurring in the plane perpendicular to that of the perturbation demonstrate that human balance control is a three-dimensional problem. Though a disturbance might occur only in the frontal plane or only in the sagittal plane, both planes remain linked in time as there is only a single instance of foot placement. Furthermore, a subject might have to compromise between the mediolateral and the anterior distance to the leading foot at foot contact, due to the finite length of our legs. A hip abduction or adduction might therefore require a paired change in hip flexion or extension, for example to ensure that wider steps are also steps of shorter length^2^. Consequently, findings in two-dimensional models of walking are not guaranteed to successfully transfer to the three-dimensional case. For example, it was previously hypothesized that a neuromuscular controller used to mimic the sagittal-plane biomechanics of human walking could also drive a three-dimensional model^29^. Though the experimental data-based controller indeed assisted in the ML direction, additional mechanisms were required to contribute to frontal plane balance. Simulations and prototype studies have furthermore shown that dynamic walking stability can be enhanced through motion coupling between different planes^30,31^.

The observed balance recovery responses can involve spinal and supra-spinal neural pathways. This can be deducted from the latencies observed in the muscle responses, as well as from the magnitude and direction dependency of the responses arising in multiple muscles simultaneously. The latter suggests that sensory integration is involved in generating the responses^26^, therefore likely involving supra-spinal processing.

Many muscles show a functional change in activity within 100 ms after perturbation onset. For example, sudden increases in GLM activity following outward perturbations facilitate outward leg swing. Such activity is phase dependent, and not strictly dependent on the background activity^32^. It has been proposed that humans modulate the GLM activity of their swing leg based on the distance between the COM and the stance foot during unperturbed walking^16^. Our outward perturbations lead to increases in this distance, and also lead to increases in GLM activity in the right leg during swing. However, theoretically, for a given distance between the COM and the stance foot there could be various COM velocities, potentially requiring different adjustments in lateral foot placement. It is therefore unlikely that lateral foot placement, and with it the GLM activity, depends solely on the distance between the COM and the stance foot. It also depends on the COM velocity^2,33^. Furthermore, the ab- and adductor muscles also respond to AP perturbations, suggesting that there are additional influences from movement in the direction perpendicular to the perturbation direction.

Decreases in muscle activity, such as those in the GLM and GM muscles following various perturbations, might be caused by reciprocal inhibition or an unload response. The latter was previously shown in the soleus muscle following plantar flexion perturbations^34^ or a sudden drop in ground support^35^, both during walking. Such activity decreases occur after removing the load from a muscle, suggesting a reduction in the contribution of spinally mediated force-sensitive afferents to the muscle activation. In our results, however, the reduction in activity does not modulate with the perturbation magnitude, such that an unload response is less likely.

In future studies we will use these perturbed gait data to validate control principles in walking models that aim to replicate stable human-like walking. For example, previously developed reflex-based walking models^14,15,36^ might be optimized using the collected data to not only have the muscles encode for locomotion, but also facilitate reactive balance recovery in response to large perturbations. In turn, such models can be used to drive devices that aim to provide balance and walking support, including orthoses such as lower extremity exoskeletons^37–39^, support-backpacks^40^, as well as (neuro)prostheses^41,42^. These devices try to extend balance control beyond the capabilities of the (impaired) user, and closer to that of a healthy human. Models might furthermore be used to extrapolate and predict balance responses to disturbances not present in the data, as it is a cumbersome task to experimentally map all responses to the tremendous amount of different perturbations that could occur during gait. Nevertheless, experimental data remains of crucial importance to provide steps toward understanding and mimicking human-like balance control during walking.

## Methods

For the presented analysis we used data from 9 subjects collected in Vlutters *et al*.^2^ (five men, age: 25 ± 2 years, weight: 68 ± 11 kg, height: 1.82 ± 0.08 m). The former study also provides details regarding the experimental setup and protocol. A brief description is provided below. Both the setup and protocol were approved by the local ethics committee (Medisch Ethische Toetsings commissie Twente). All subjects gave written informed consent before participation, in accordance with the Declaration of Helsinki.

### Apparatus

**Figure 9:**
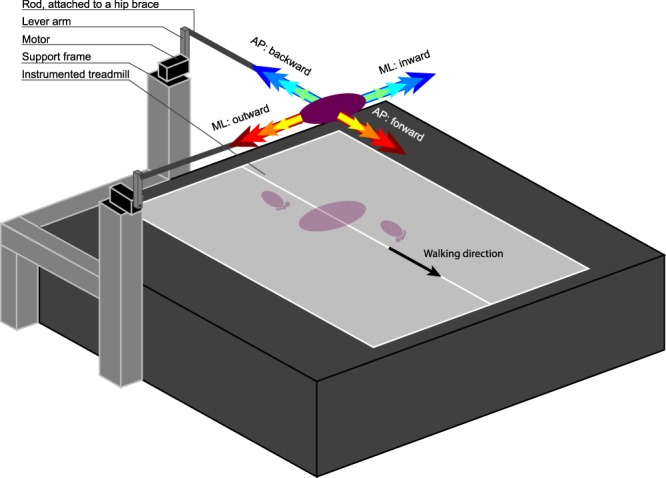
Experimental setup. Subjects walked on an instrumented treadmill, while receiving pelvis perturbations using a motor at either the right side or the rear of the treadmill. The motor was attached to the subject through a lever arm, a horizontal rod, and a brace that the subject could wear around the pelvis. The interaction force between subject and motor was kept to a minimum while no perturbation was being applied.

Subjects walked on a dual-belt instrumented treadmill (MotekForce Link, Culemborg, Netherlands), and received both ML and AP perturbations at the pelvis through a motor (Moog, Nieuw-Vennep, Netherlands), see Fig. 9. The motor could be connected to the subject’s pelvis through a horizontal aluminum rod and a hip-brace (Distrac Wellcare, Hoegaarden, Belgium). Motor control was done over Ethernet (User Datagram Protocol) using xPC-target (MathWorks, Natick, MA, USA) with a dedicated network card (82558 Ethernet card, Intel, Santa Clara, CA, USA). The same xPC-target was used to collect 3 degrees-of-freedom ground reaction forces and moments of each of the two force plates in the treadmill, using a PCI-6229 AD card (National Instruments, Austin, TX, USA). A Bagnoli Desktop system (Delsys Inc, Natick, MA, USA) with an internal bandpass filter (20–450 Hz) was used to collect the bilateral EMG activity of the tibialis anterior (TA), gastrocnemius medialis (GM), rectus femoris (RF), biceps femoris (BF), gluteus medius (GLM), and adductor longus (AL) muscles. These EMG signals were digitized using the PCI card. All motor, force plate, and EMG signals were collected at 1 kHz. Finally, a 12-camera motion capture system (Visualeyez-II, Phoenix Technologies Inc, Burnaby, Canada) and LED clusters were used to collect kinematic data of the subject’s feet, lower legs, upper legs, pelvis, trunk, and head. All kinematic data were collected at 100 Hz.

### Protocol

Prior to the experiment, bony landmarks were captured using an LED based probe, relative to the LED clusters on each segment. Landmarks were the calcaneus, 1^st^ and 5^th^ metatarsal heads, medial and lateral malleoli, fibula head, medial and lateral epicondyles of the femur, greater trochanter, anterior and posterior superior iliac spines, xiphoid process, jugular notch, seventh cervical vertebra, occiput, head vertex and nasal sellion^43^.

Next, subjects were asked to walk on the treadmill with their arms crossed over the abdomen. Each subject walked four blocks of three trials each. In each block, the first trial was an unperturbed walking trial, while the second and third were perturbation trials. In two blocks only ML perturbations were applied, and in two blocks only AP perturbations were applied. For each direction (ML or AP), subjects walked one block at a slow speed (2.25 km/h) and one at a normal speed (4.5 km/h), with each speed scaled to the square root of the subject’s leg length^22^ to normalize to leg length. During perturbation trials, perturbation onset could randomly occur at the instance of TOR, detected using the vertical ground reaction force. Toe-off was chosen as perturbation onset to provide subjects with the maximum amount of time to recover balance through foot placement adjustments, while preventing immediate modulation of the push-off in reaction to the disturbances. Perturbations were implemented as 150 ms block pulses of force magnitude equal to 4, 8, 12, and 16% of the subject’s body weight. For ML perturbations, the direction of the force could be inward (leftward for right swing leg) or outward (rightward for right swing leg). With this definition, inward perturbations during the swing phase generally lead to a first recovery step that is also inward (i.e. toward the mid-line of the body), whereas outward perturbations generally lead to a first recovery step outward. For AP perturbations, the directions were forward and backward. Each combination of perturbation direction, magnitude, and walking speed was repeated 8 times, yielding 256 perturbations per subject. When no perturbation was applied, the motors were admittance controlled such that the interaction force between subject and motor was as small as possible (reflected inertia <1 kg).

### Data processing

Data were processed using Matlab (R2016b, Mathworks) and OpenSim v3.3^44^. In Matlab, marker data were filtered using a 4^nd^ order 20 Hz zero-phase low-pass Butterworth filter before reconstructing the bony landmark positions through singular value decomposition^45^. The same filter was applied to the ground reaction forces and moments, which were subsequently resampled to 100 Hz to match the marker data. Finally, the EMG recordings were filtered using a 1^st^ order 48–52 Hz Butterworth bandstop filter, then detrended by subtracting the signal average, rectified, and filtered with a 2^nd^ order 20 Hz low-pass Butterworth filter.

In OpenSim, the default 23 degrees-of-freedom gait2354 model was used for the inverse kinematics and inverse dynamics. In this model the arms are assumed to be kept over the chest, and the head and trunk together make up one segment. For each subject, the model was scaled to subject dimensions using the landmark data. Joint angles were calculated using inverse kinematics, by matching the model markers to those in the recordings. The head marker data were not used in the inverse kinematics, as the head is part of the trunk segment in the model. The inverse kinematics results were filtered with OpenSim’s default 6 Hz low-pass filter prior to the inverse dynamics analysis. In the inverse dynamics, the ground reaction forces and moments recorded using the left and right force plates were applied to the left and right calcaneus segments of the model, respectively. Furthermore, the measured force exerted by the motor on the subject was modeled as a horizontal force applied to the center of mass of the model’s pelvis.

In Matlab, for each subject the joint angles from a static measurement were subtracted from all kinematic data, such that the subject’s upright standing position corresponded to joint angles of 0 radians. Joint power was calculated by multiplying the joint velocities with the joint moments. Joint moments and power were subsequently made dimensionless following^22^ and^23^, respectively. The EMG data of each muscle was scaled to the maximum activity of that muscle occurring within the median of the activity over all unperturbed gait cycles. For each muscle of each subject, we furthermore located the onset instances of changes in EMG activity as a result of the perturbations. This onset instance was taken as the first instant in time following perturbation onset at which the perturbed EMG data deviated from the unperturbed EMG data with more than three times the repetition standard deviation of the unperturbed EMG. These onset instances were subsequently averaged over repetitions, and subjects.

Next, gait phases were detected using landmarks on the feet, in line with^46^. Using the instances of perturbation onset at TOR, perturbation end, and the gait events of toe-off left and right (TOL, TOR, respectively), and heel strike left and right (HSL, HSR, respectively), all data were cut into sequences, together spanning one gait cycle after perturbation onset. The duration of each of these sequences was determined, before resampling each sequence to 50 samples. For each subject, all sequences and their durations were sorted on perturbation magnitude and direction. These were then averaged over the 8 repetitions per condition, to obtain within-subject averages and standard deviations. In turn, these data were averaged over all subjects.

Finally, linear mixed models were used to assess the effect of the perturbation magnitude (fixed factor, with intercept) on various points of interest in the data. This was done merely to check whether the changes at the observed points of interest in the data were not simply caused by subject variability. Tests were performed separately for the four perturbation directions. To account for correlation effects from repeated measures within the same subject, subject effects were included as a random factor (intercept). A significance level of α = 0.05 was used. Only main effects were considered, and no *post-hoc* analysis was performed. SPSS statistics 21 (IBM Corporation, Armonk, NY, USA) was used for the statistical analysis.

## Electronic supplementary material

Supplementary Figures

Supplementary Dataset

## Data Availability

Additional figures and corresponding data are available in the Supplementary Information.
