# Supplementary material for: Lower extremity joint-level responses to pelvis perturbation during human walking
Source: Sci Rep. 2018 Oct 2;8:14621. doi: 10.1038/s41598-018-32839-8 (PMC6168500; doi:10.1038/s41598-018-32839-8)
Supplement: Supplementary file 1 — Supplementary Figures [file 41598_2018_32839_MOESM1_ESM.pdf]

Supplementary material to:

Lower extremity joint-level responses to pelvis perturbations during human walking

M. Vlutters, E.H.F. van Asseldonk, H. van der Kooij

For completion, the figures resulting from the inverse kinematics and inverse dynamics analysis are presented in these supplementary materials. In all figures, data is shown for one gait cycle after perturbation onset at toe-off right (TOR), split into sequences. Angles are in radians. Moment and power are presented dimensionless, with subject average scaling factors of 1215±259 Nm and 2739±562 W, respectively. Colors indicate the different perturbations. Yellow-red corresponds with outward perturbations (ML) or forward perturbations (AP), green-blue with inward perturbations (ML) or backward perturbations (AP). The gray shading is the subject-average standard deviation, which is only shown for the unperturbed condition to prevent image cluttering. In case of inward perturbations, joint moments and power are left out due to cross-stepping, which resulted in subjects standing with both feet on the same force plate. The horizontal length of each line in a sequence reflects the duration of that sequence relative to the other conditions.

Figure S1 – S7 : slow walking (2.25 km/h), mediolateral perturbations
Figure S8 – S14 : slow walking (2.25 km/h), anteroposterior perturbations
Figure S15 – S21 : normal walking (4.50 km/h), mediolateral perturbations
Figure S22 – S28 : normal walking (4.50 km/h), anteroposterior perturbations

Fig. S1

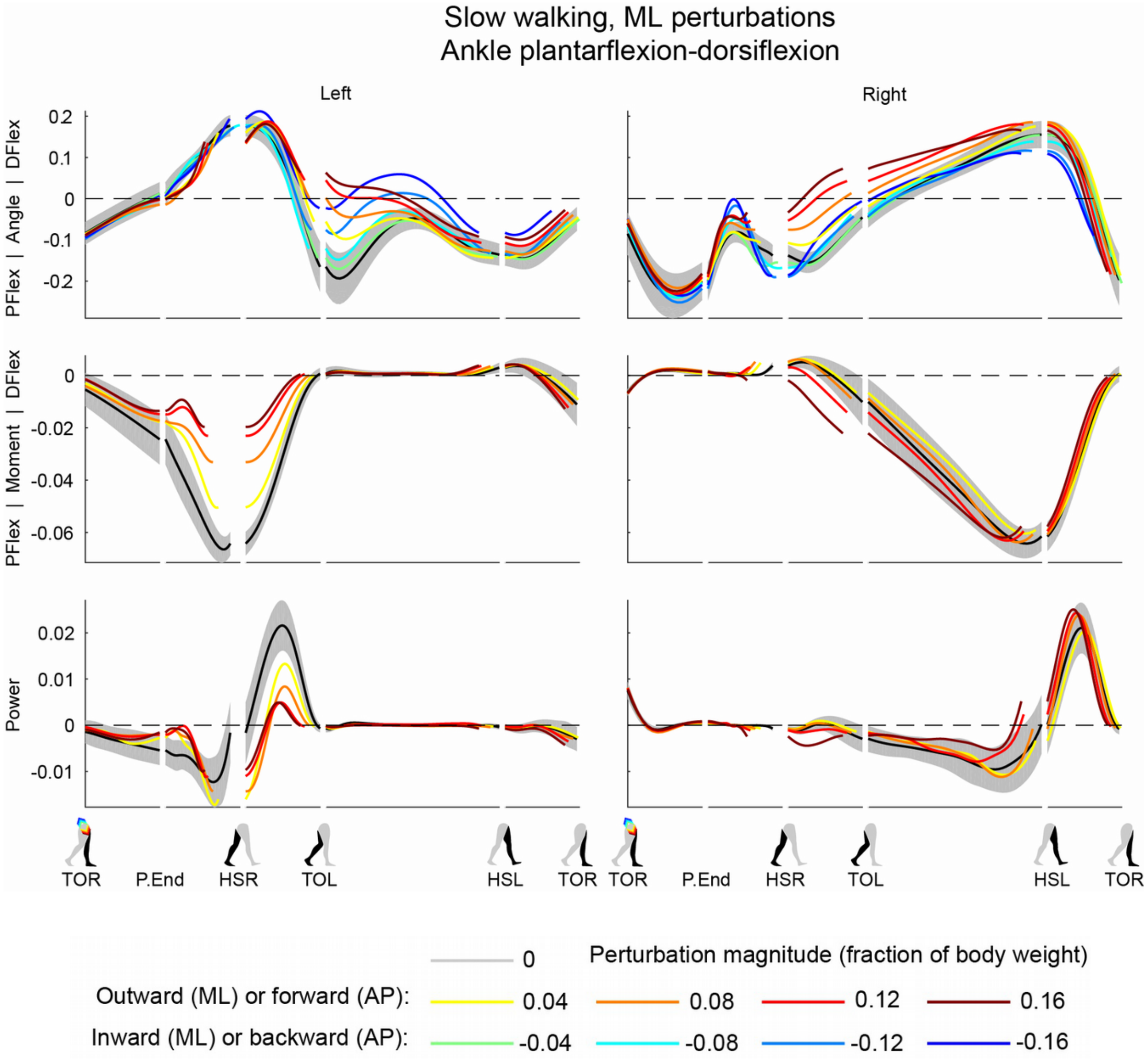

Slow walking, ML perturbations
Ankle plantarflexion-dorsiflexion
Left
Right
PFlex | Angle | DFlex
0.2
0.1
0
-0.1
-0.2
PFlex | Moment | DFlex
0
-0.02
-0.04
-0.06
Power
0.02
0.01
0
-0.01
TOR
P.End
HSR
TOL
HSL
TOR
TOR
P.End
HSR
TOL
HSL
TOR
0
Perturbation magnitude (fraction of body weight)
Outward (ML) or forward (AP):
0.04
0.08
0.12
0.16
Inward (ML) or backward (AP):
-0.04
-0.08
-0.12
-0.16

Fig. S2

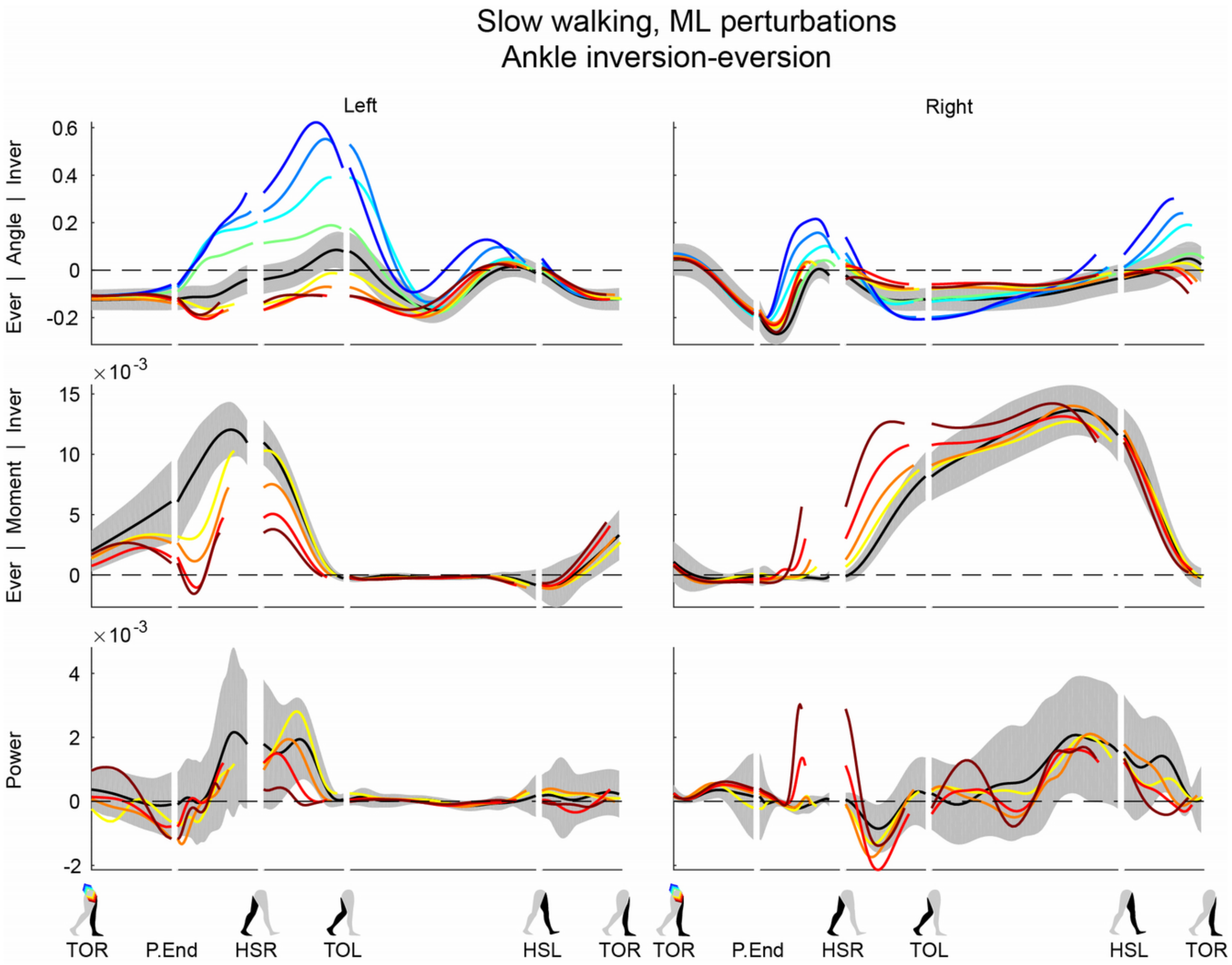

Slow walking, ML perturbations
Ankle inversion-eversion
Left
Right
Ever | Angle | Inver
0.6
0.4
0.2
0
-0.2
×10^-3
Ever | Moment | Inver
15
10
5
0
×10^-3
Power
4
2
0
-2
TOR
P.End
HSR
TOL
HSL
TOR
TOR
P.End
HSR
TOL
HSL
TOR

Fig. S3

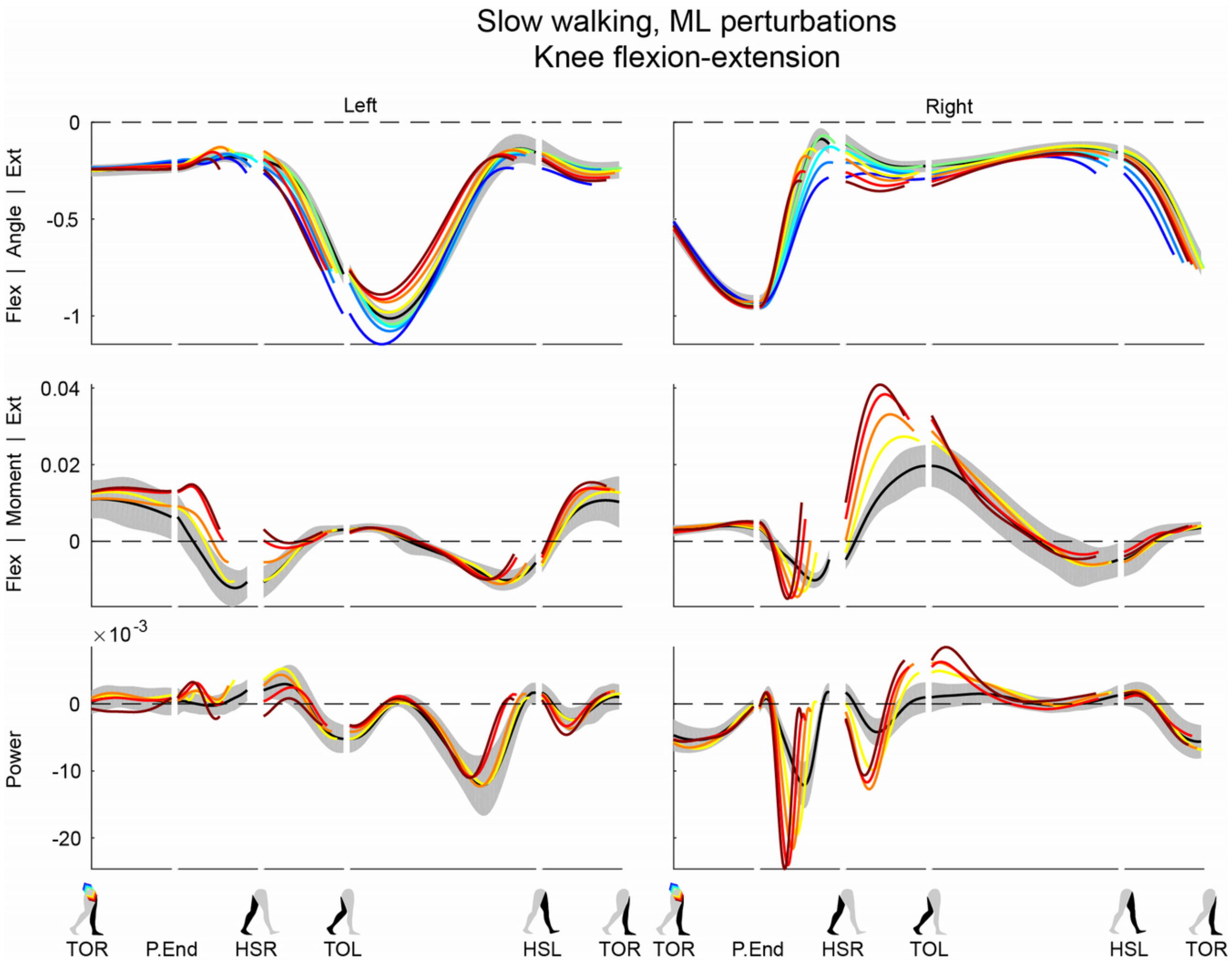

Slow walking, ML perturbations
Knee flexion-extension
Left
Right
Flex | Angle | Ext
0
-0.5
-1
Flex | Moment | Ext
0.04
0.02
0
Power
×10^-3
0
-10
-20
TOR
P.End
HSR
TOL
HSL
TOR
TOR
P.End
HSR
TOL
HSL
TOR

Fig. S4

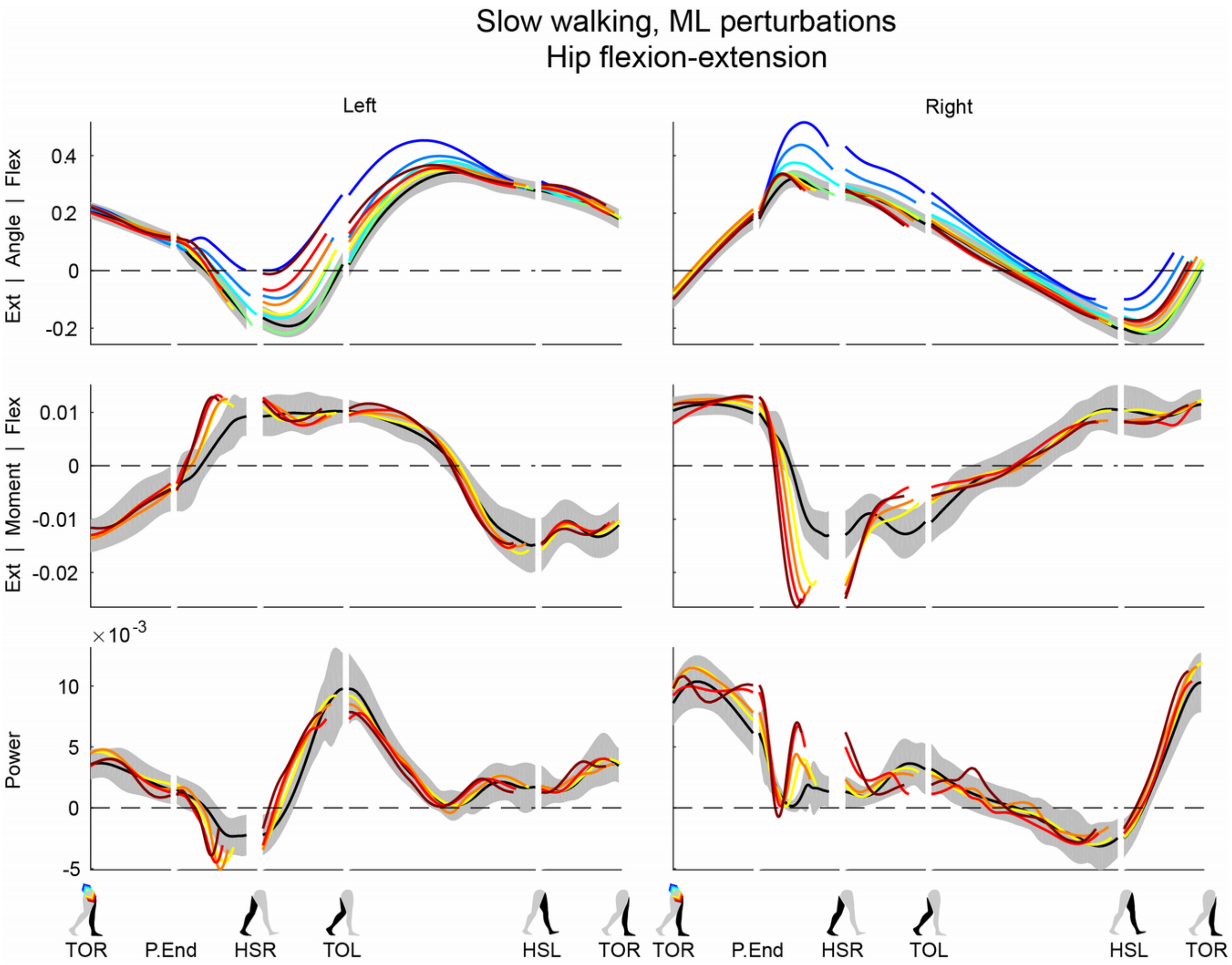

Slow walking, ML perturbations
Hip flexion-extension
Left
Right
Ext | Angle | Flex
0.4
0.2
0
-0.2
Ext | Moment | Flex
0.01
0
-0.01
-0.02
×10^-3
Power
10
5
0
-5
TOR
P.End
HSR
TOL
HSL
TOR
TOR
P.End
HSR
TOL
HSL
TOR

Fig. S5

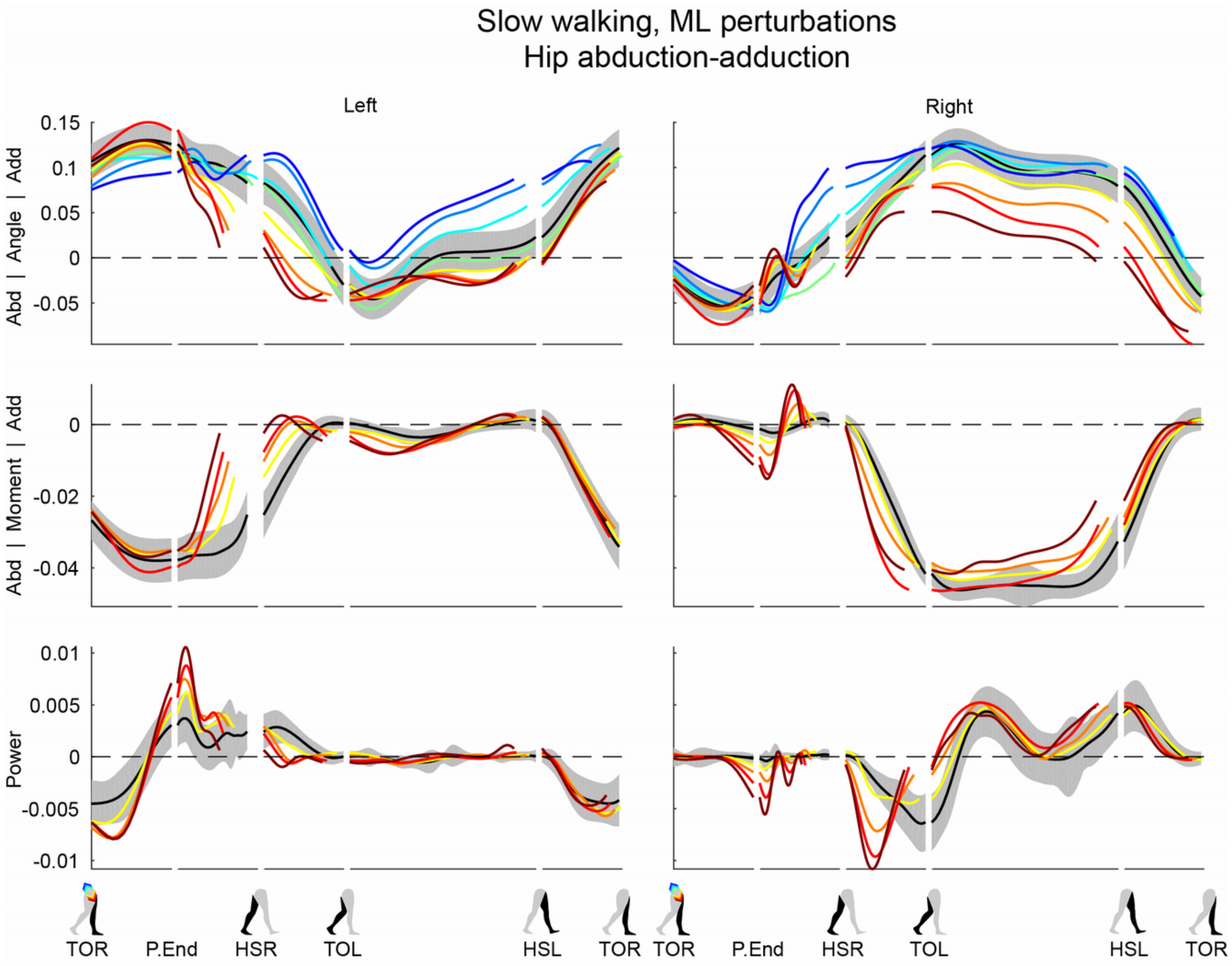
Slow walking, ML perturbations
Hip abduction-adduction
Left
Right
Abd | Angle | Add
0.15
0.1
0.05
0
-0.05
Abd | Moment | Add
0
-0.02
-0.04
Power
0.01
0.005
0
-0.005
-0.01
TOR
P.End
HSR
TOL
HSL
TOR
TOR
P.End
HSR
TOL
HSL
TOR

Fig. S6

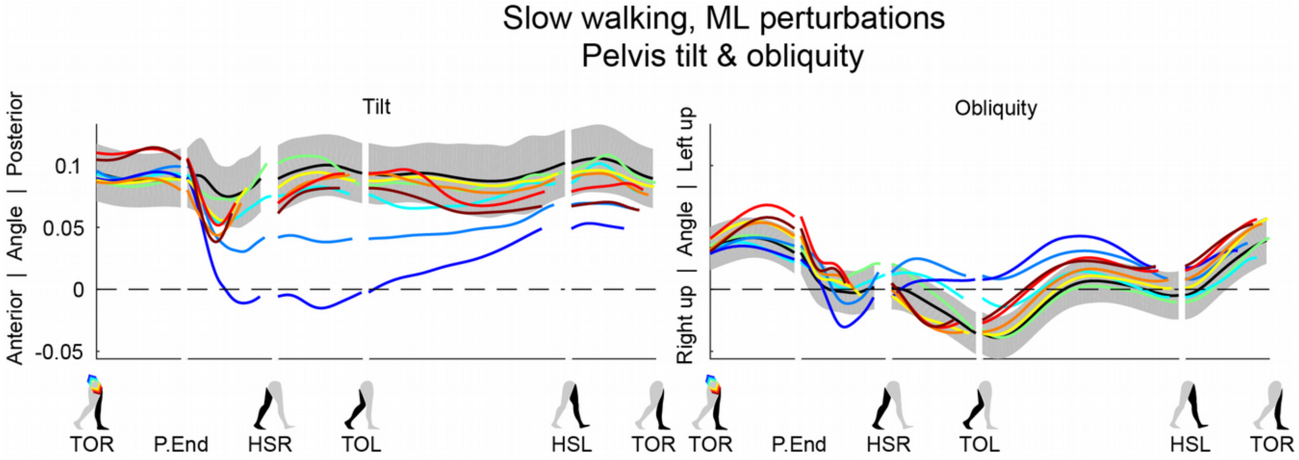
Slow walking, ML perturbations
Pelvis tilt & obliquity
Tilt
Anterior | Angle | Posterior
0.1
0.05
0
-0.05
TOR
P.End
HSR
TOL
HSL
TOR
Obliquity
Right up | Angle | Left up
TOR
P.End
HSR
TOL
HSL
TOR

Fig. S7

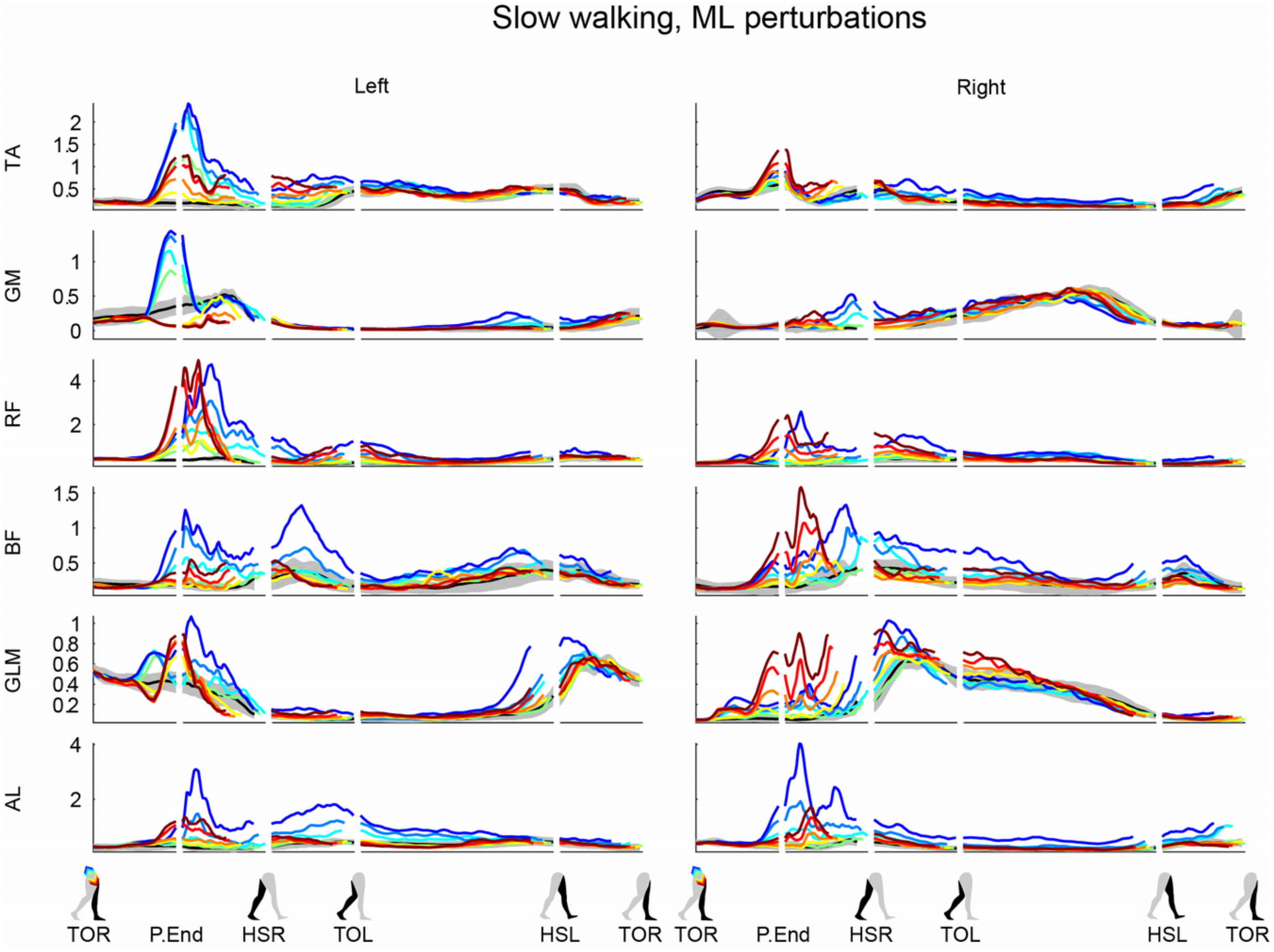
Slow walking, ML perturbations
Left
Right
TA
GM
RF
BF
GLM
AL
TOR
P.End
HSR
TOL
HSL
TOR
TOR
P.End
HSR
TOL
HSL
TOR

Fig. S8

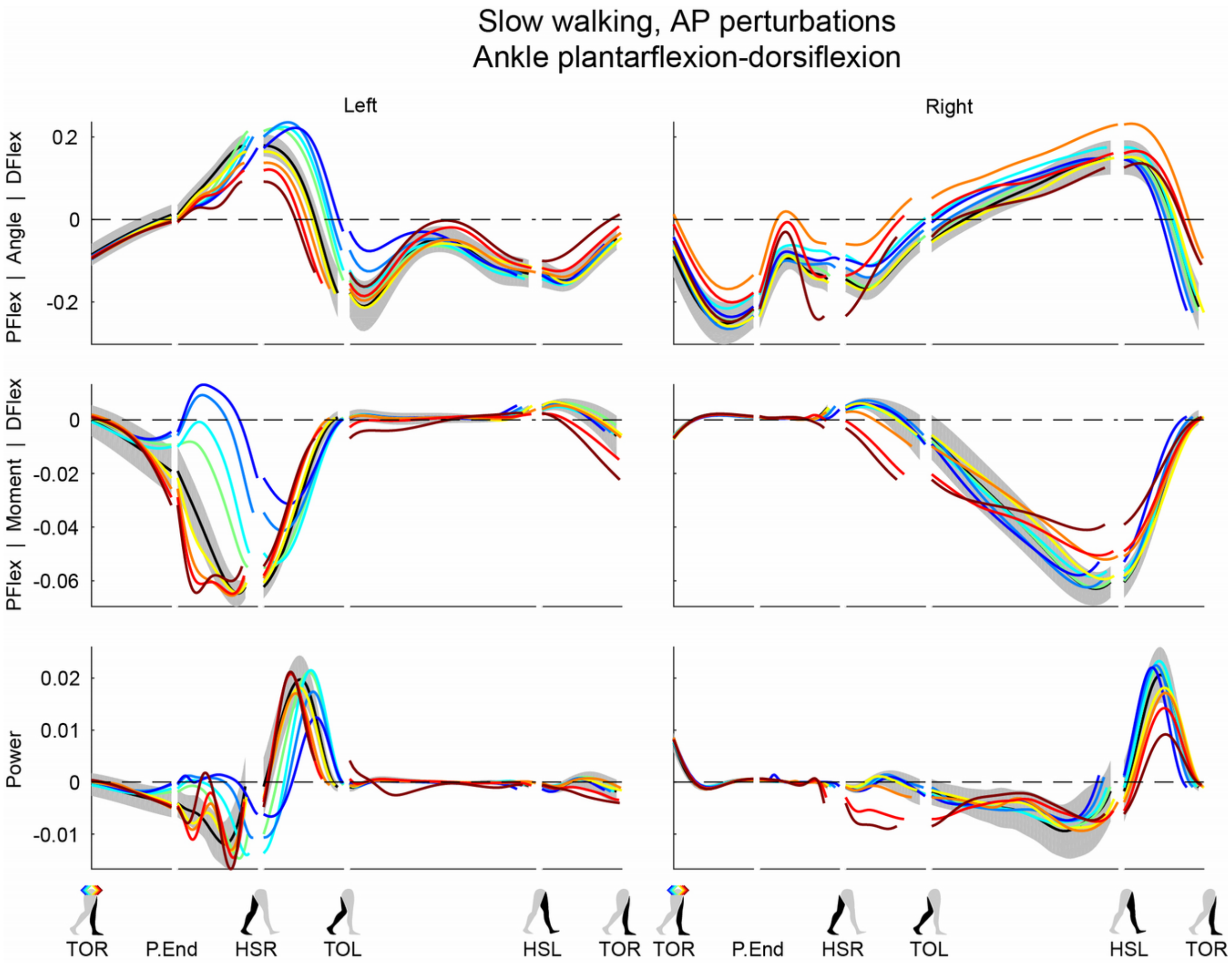
Slow walking, AP perturbations
Ankle plantarflexion-dorsiflexion
Left
Right
PFlex | Angle | DFlex
0.2
0
-0.2
PFlex | Moment | DFlex
0
-0.02
-0.04
-0.06
Power
0.02
0.01
0
-0.01
TOR
P.End
HSR
TOL
HSL
TOR
TOR
P.End
HSR
TOL
HSL
TOR

Fig. S9

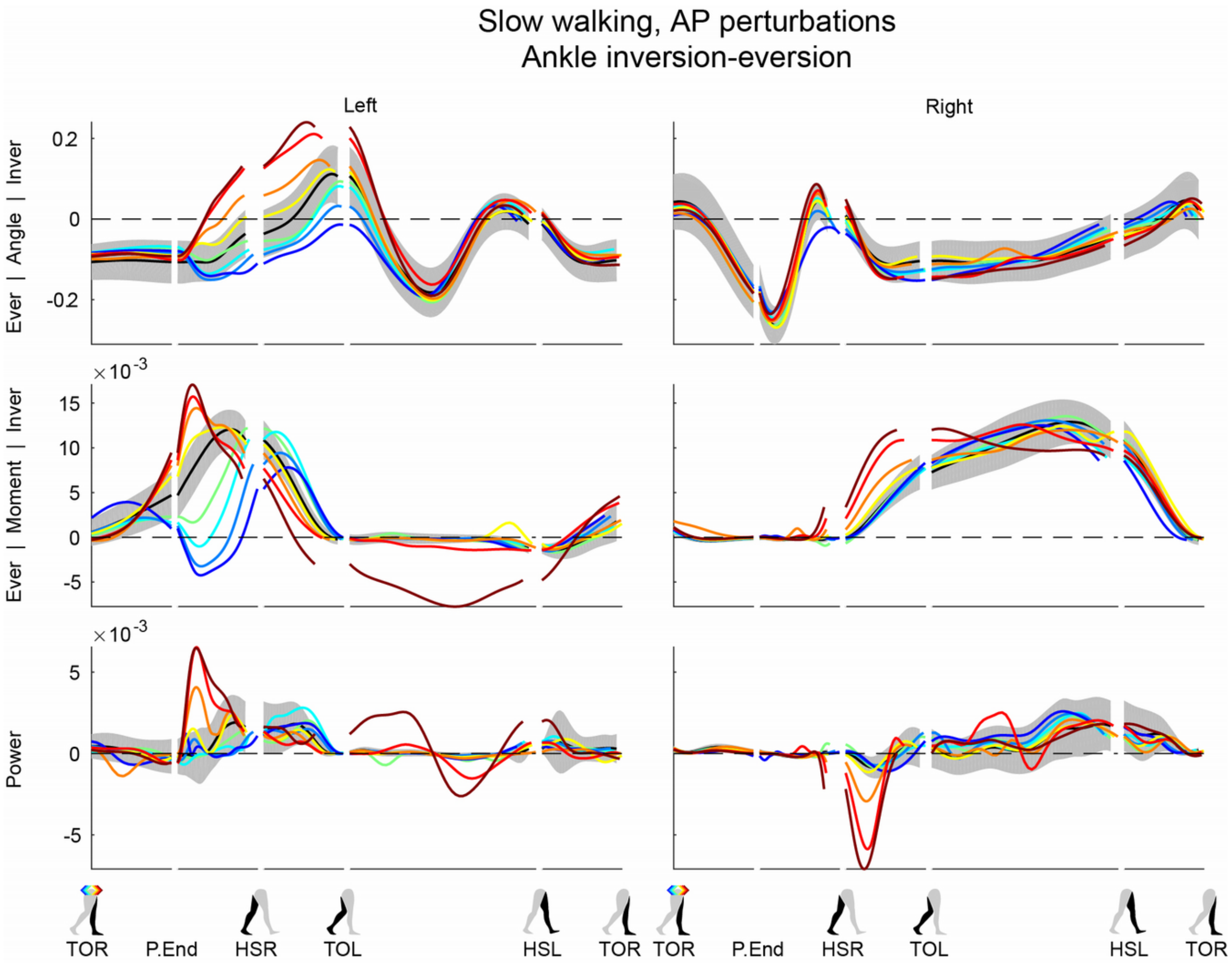
Slow walking, AP perturbations
Ankle inversion-eversion
Left
Right
Ever | Angle | Inver
0.2
0
-0.2
×10-3
Ever | Moment | Inver
15
10
5
0
-5
×10-3
Power
5
0
-5
TOR
P.End
HSR
TOL
HSL
TOR
TOR
P.End
HSR
TOL
HSL
TOR

Fig. S10

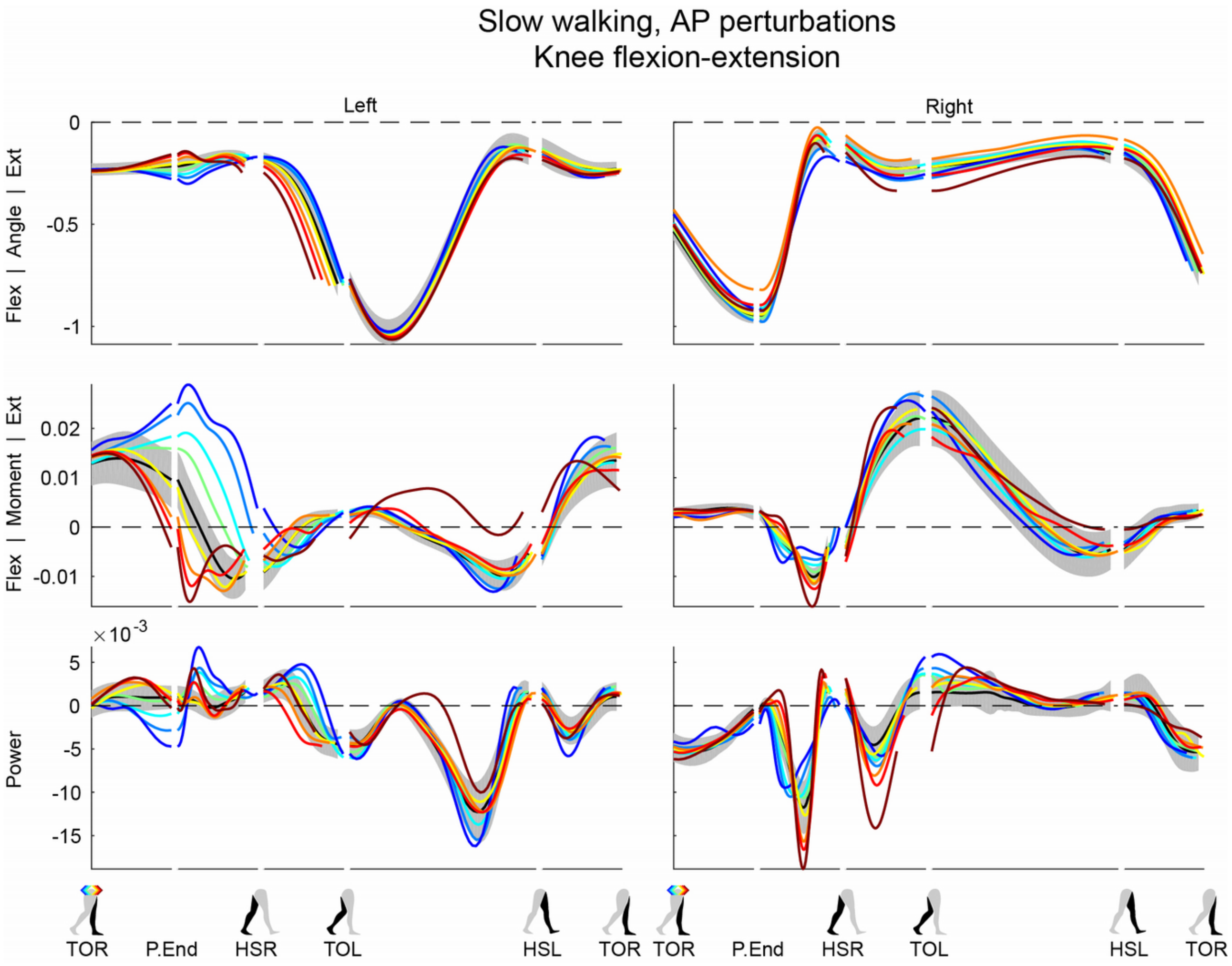

Slow walking, AP perturbations
Knee flexion-extension
Left
Right
Flex | Angle | Ext
Flex | Moment | Ext
Power
0
-0.5
-1
0.02
0.01
0
-0.01
$\times 10^{-3}$
5
0
-5
-10
-15
TOR
P.End
HSR
TOL
HSL
TOR
TOR
P.End
HSR
TOL
HSL
TOR

Fig. S11

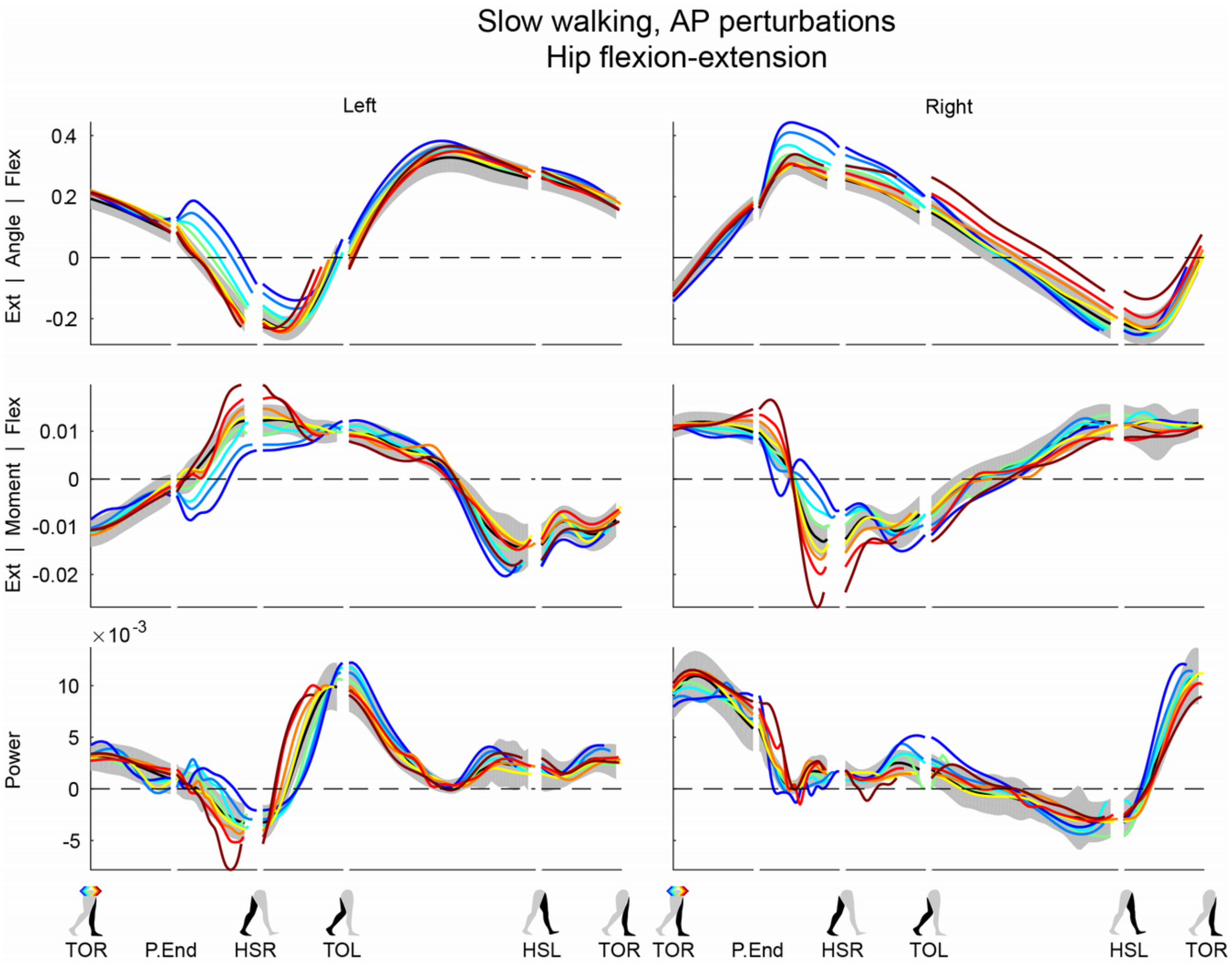

Slow walking, AP perturbations
Hip flexion-extension
Left
Right
Ext | Angle | Flex
0.4
0.2
0
-0.2
Ext | Moment | Flex
0.01
0
-0.01
-0.02
Power
×10^-3
10
5
0
-5
TOR
P.End
HSR
TOL
HSL
TOR
TOR
P.End
HSR
TOL
HSL
TOR

Fig. S12

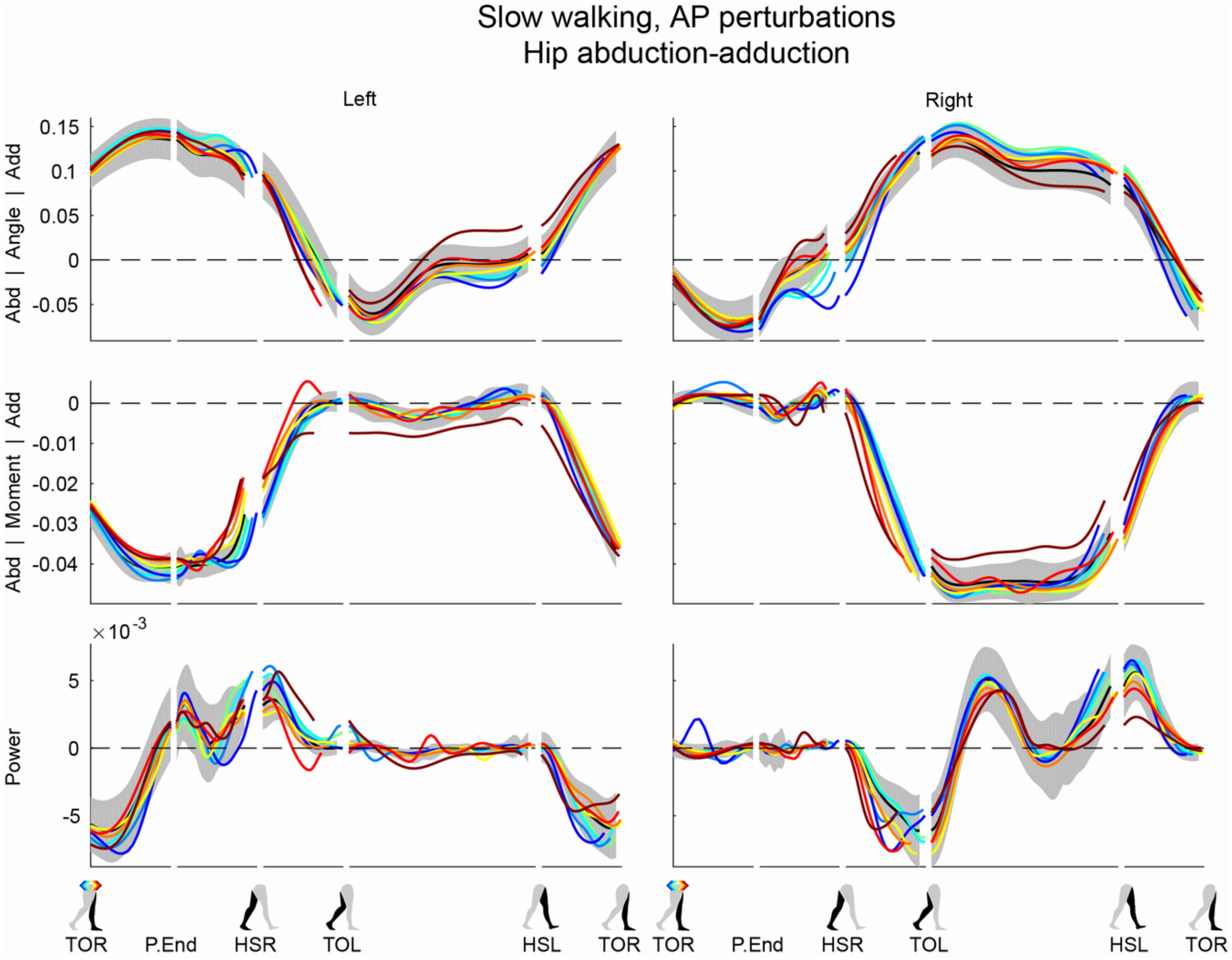

Slow walking, AP perturbations
Hip abduction-adduction
Left
Right
Abd | Angle | Add
0.15
0.1
0.05
0
-0.05
Abd | Moment | Add
0
-0.01
-0.02
-0.03
-0.04
Power
×10^-3
5
0
-5
TOR
P.End
HSR
TOL
HSL
TOR
TOR
P.End
HSR
TOL
HSL
TOR

Fig. S13

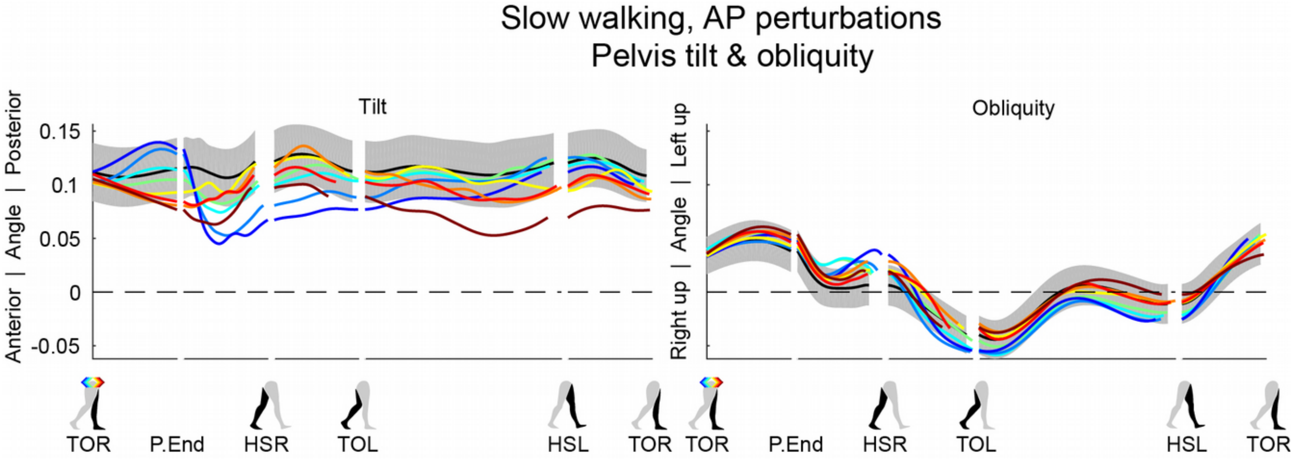

Slow walking, AP perturbations
Pelvis tilt & obliquity
Tilt
Obliquity
Anterior | Angle | Posterior
Right up | Angle | Left up
0.15
0.1
0.05
0
-0.05
TOR
P.End
HSR
TOL
HSL
TOR
TOR
P.End
HSR
TOL
HSL
TOR

Fig. S14

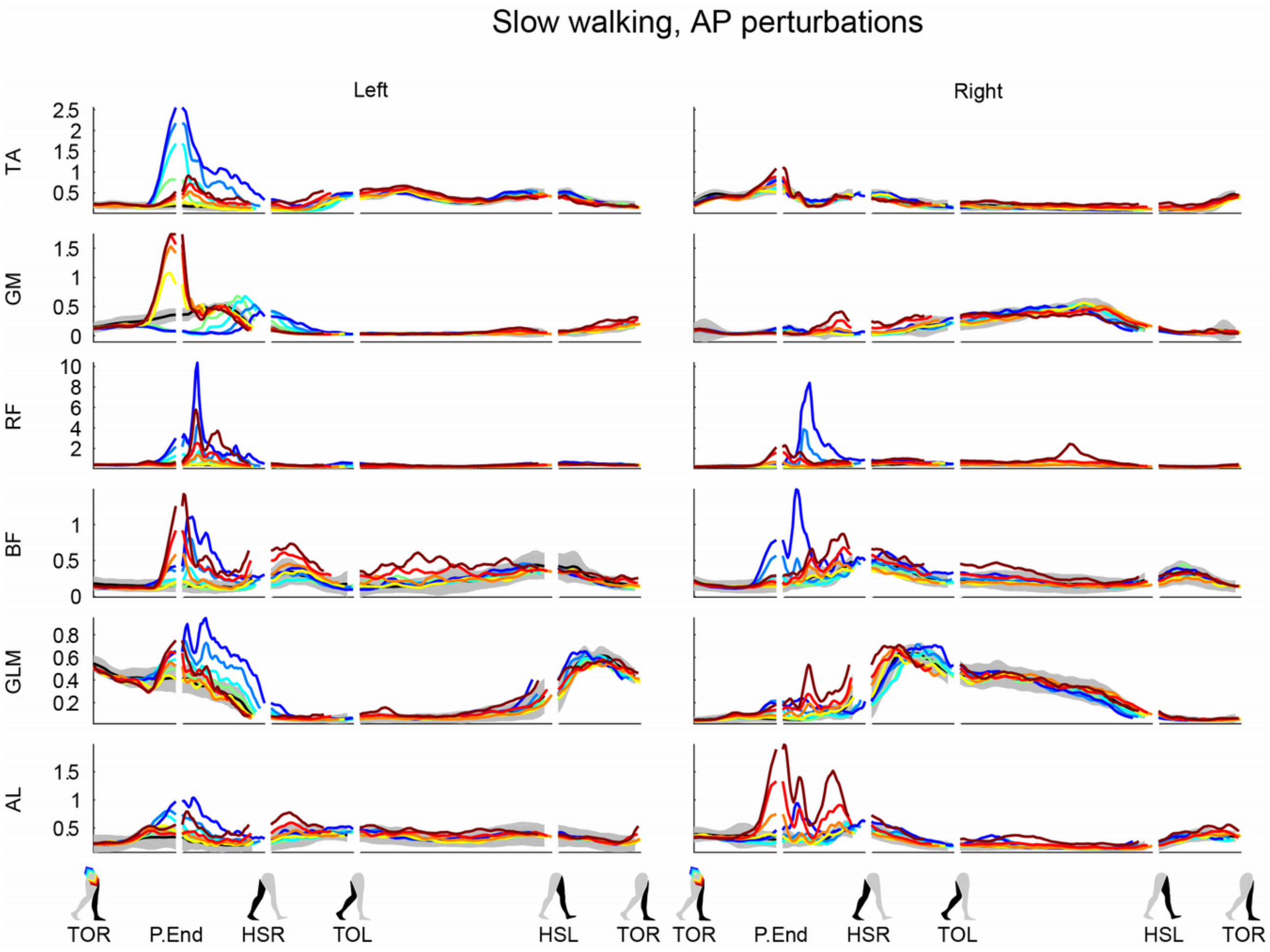

Slow walking, AP perturbations
Left
Right
TA
GM
RF
BF
GLM
AL
2.5
2
1.5
1
0.5
0
10
8
6
4
0.8
0.6
0.4
0.2
TOR
P.End
HSR
TOL
HSL
TOR
TOR
P.End
HSR
TOL
HSL
TOR

Fig. S15

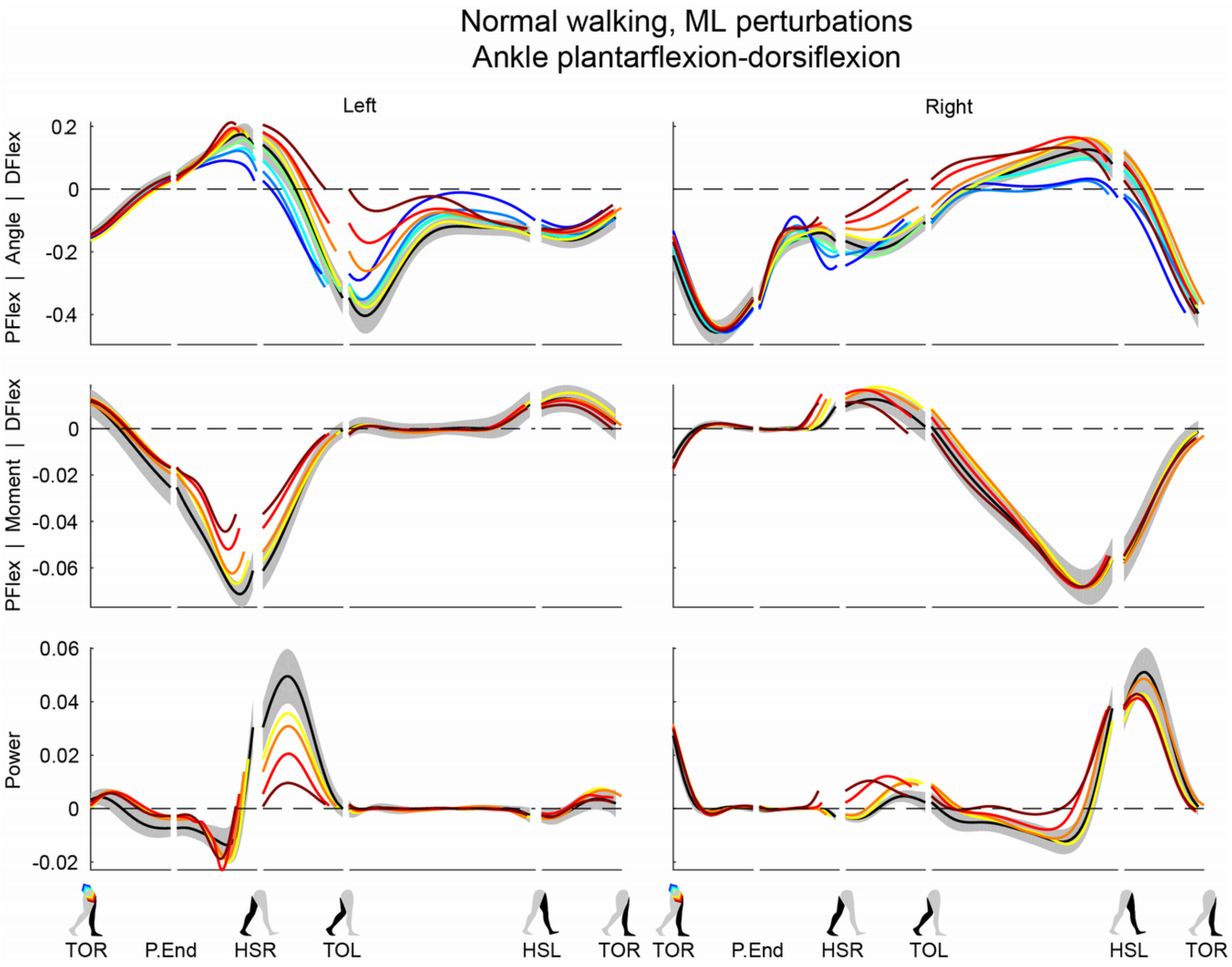
Normal walking, ML perturbations
Ankle plantarflexion-dorsiflexion
Left
Right
PFlex | Angle | DFlex
0.2
0
-0.2
-0.4
PFlex | Moment | DFlex
0
-0.02
-0.04
-0.06
Power
0.06
0.04
0.02
0
-0.02
TOR
P.End
HSR
TOL
HSL
TOR
TOR
P.End
HSR
TOL
HSL
TOR

Fig. S16

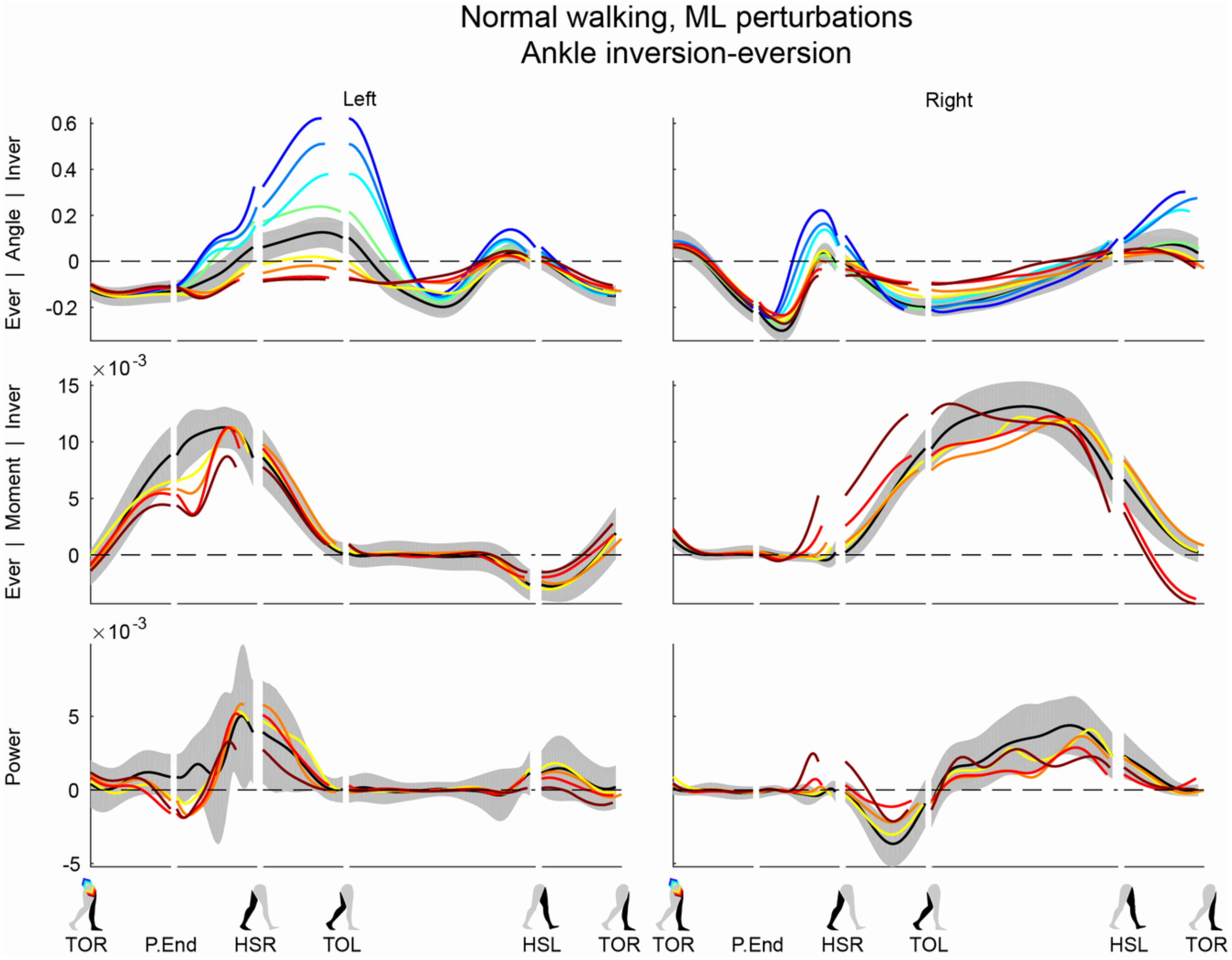

Normal walking, ML perturbations
Ankle inversion-eversion
Left
Right
Ever | Angle | Inver
0.6
0.4
0.2
0
-0.2
×10^-3
15
10
5
0
Ever | Moment | Inver
×10^-3
5
0
-5
Power
TOR
P.End
HSR
TOL
HSL
TOR
TOR
P.End
HSR
TOL
HSL
TOR

Fig. S17

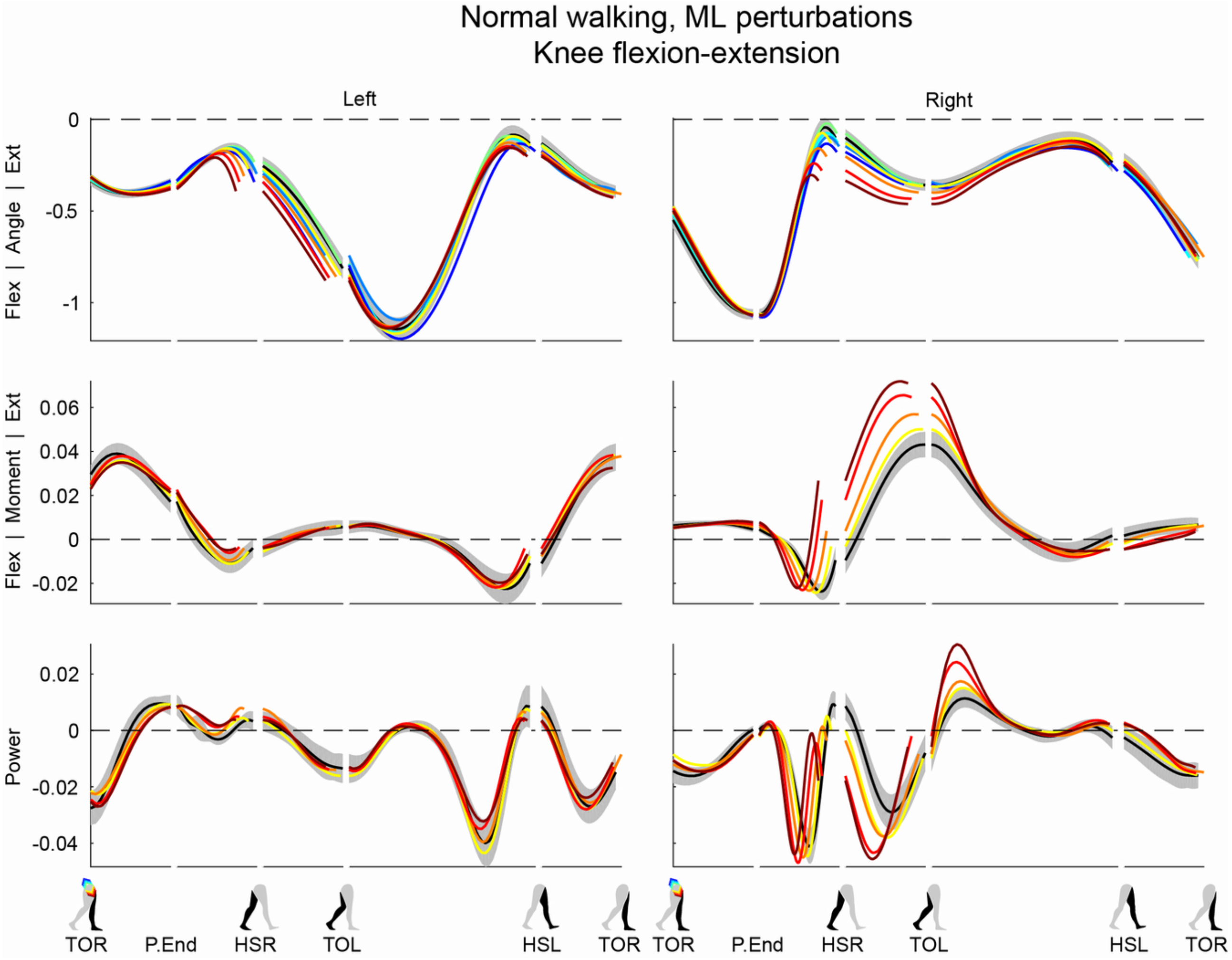

Normal walking, ML perturbations
Knee flexion-extension
Left
Right
Flex | Angle | Ext
0
-0.5
-1
Flex | Moment | Ext
0.06
0.04
0.02
0
-0.02
Power
0.02
0
-0.02
-0.04
TOR
P.End
HSR
TOL
HSL
TOR
TOR
P.End
HSR
TOL
HSL
TOR

Fig. S18

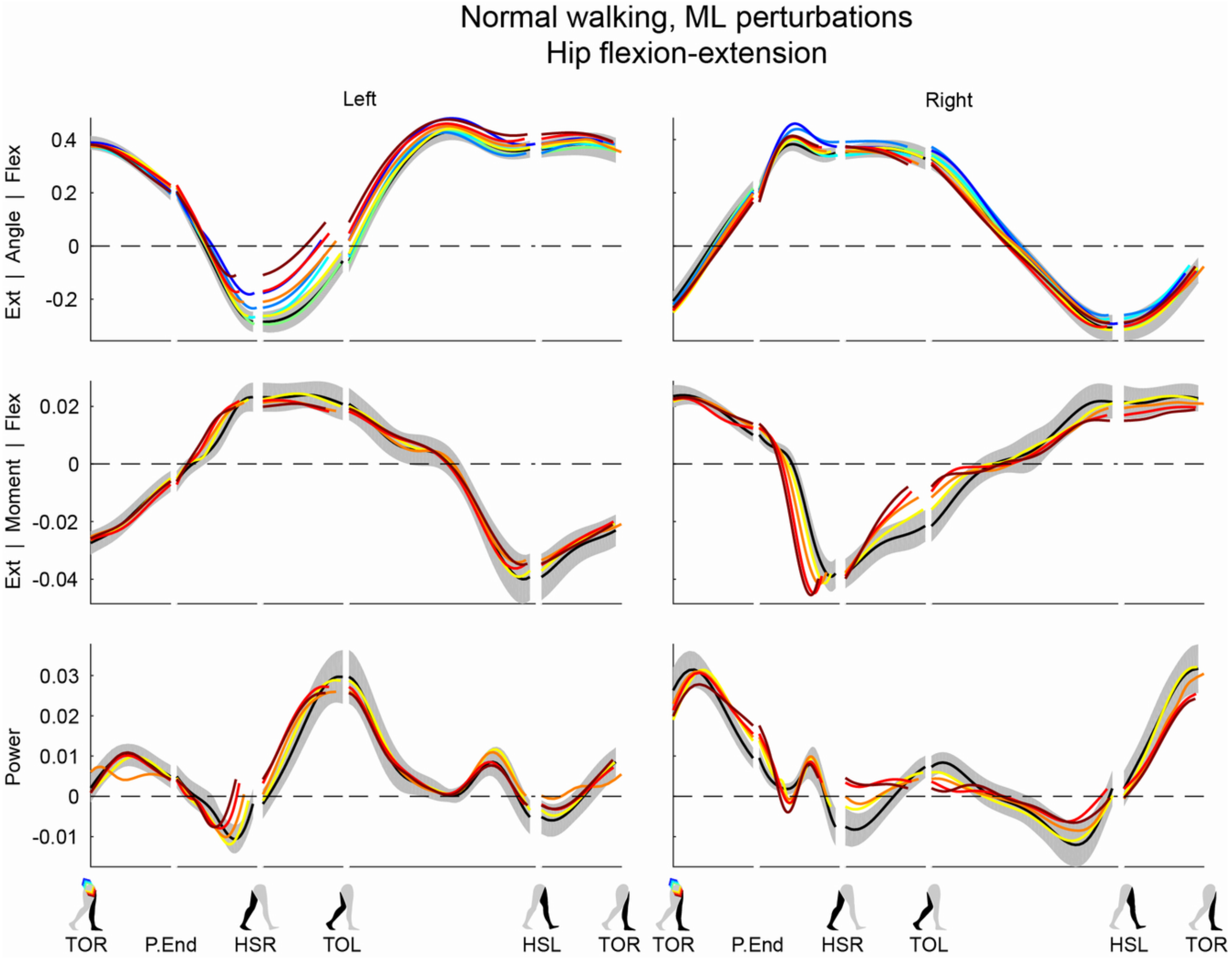

Normal walking, ML perturbations
Hip flexion-extension
Left
Right
Ext | Angle | Flex
0.4
0.2
0
-0.2
Ext | Moment | Flex
0.02
0
-0.02
-0.04
Power
0.03
0.02
0.01
0
-0.01
TOR
P.End
HSR
TOL
HSL
TOR
TOR
P.End
HSR
TOL
HSL
TOR

Fig. S19

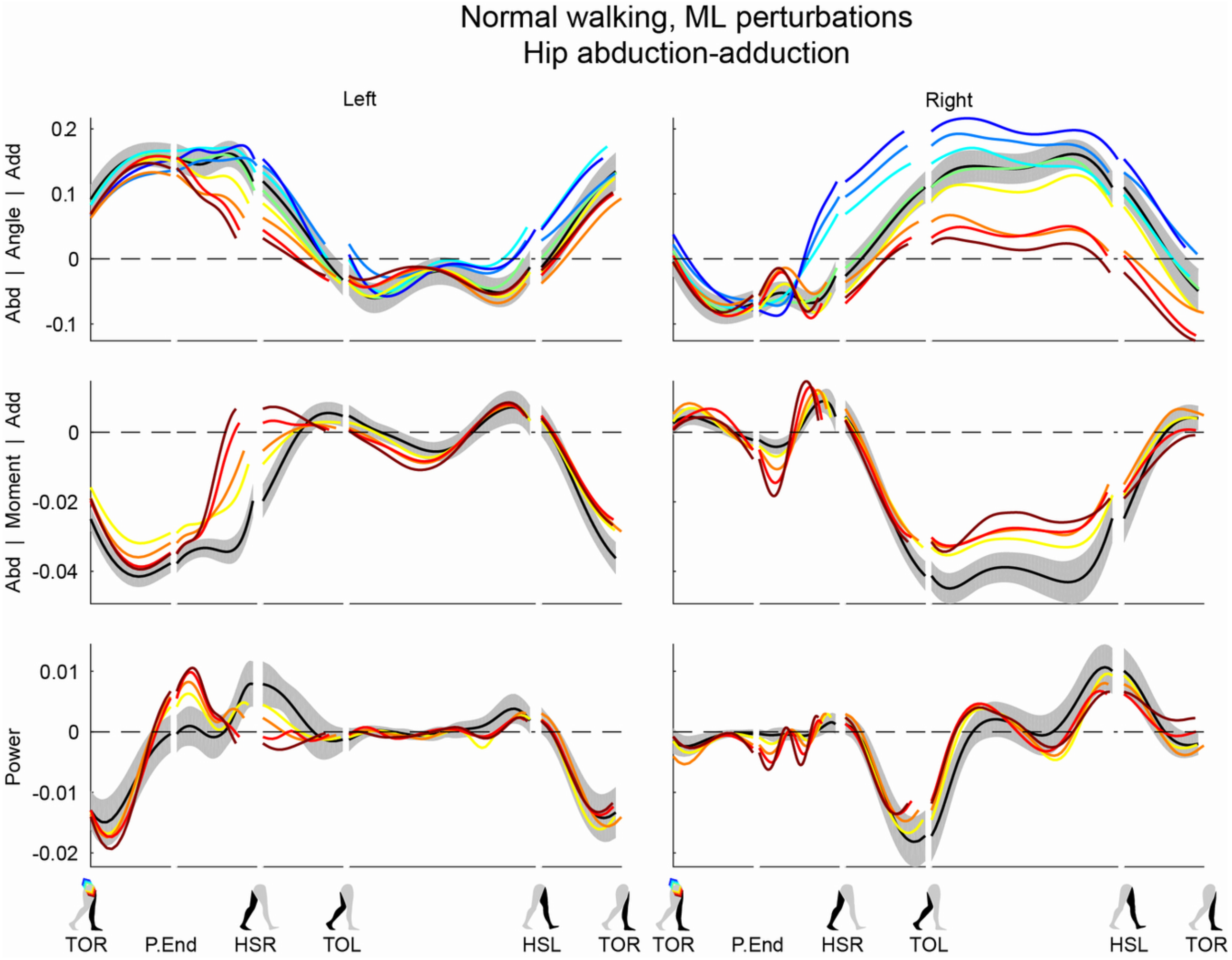

Normal walking, ML perturbations
Hip abduction-adduction
Left
Right
Abd | Angle | Add
Abd | Moment | Add
Power
0.2
0.1
0
-0.1
0
-0.02
-0.04
0.01
0
-0.01
-0.02
TOR
P.End
HSR
TOL
HSL
TOR
TOR
P.End
HSR
TOL
HSL
TOR

Fig. S20

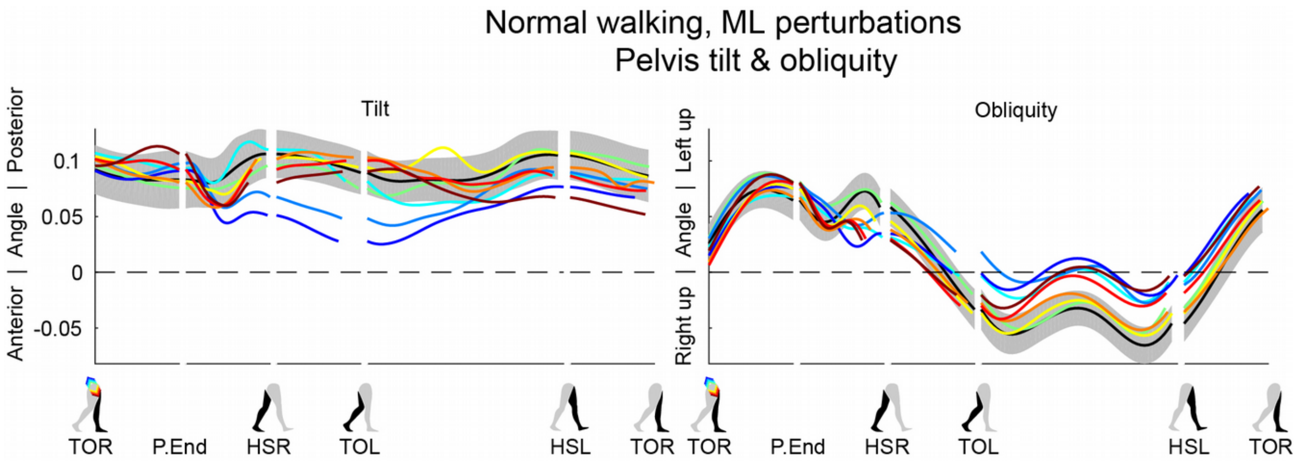
Normal walking, ML perturbations
Pelvis tilt & obliquity
Tilt
Obliquity
Anterior | Angle | Posterior
Right up | Angle | Left up
0.1
0.05
0
-0.05
TOR
P.End
HSR
TOL
HSL
TOR
TOR
P.End
HSR
TOL
HSL
TOR

Fig. S21

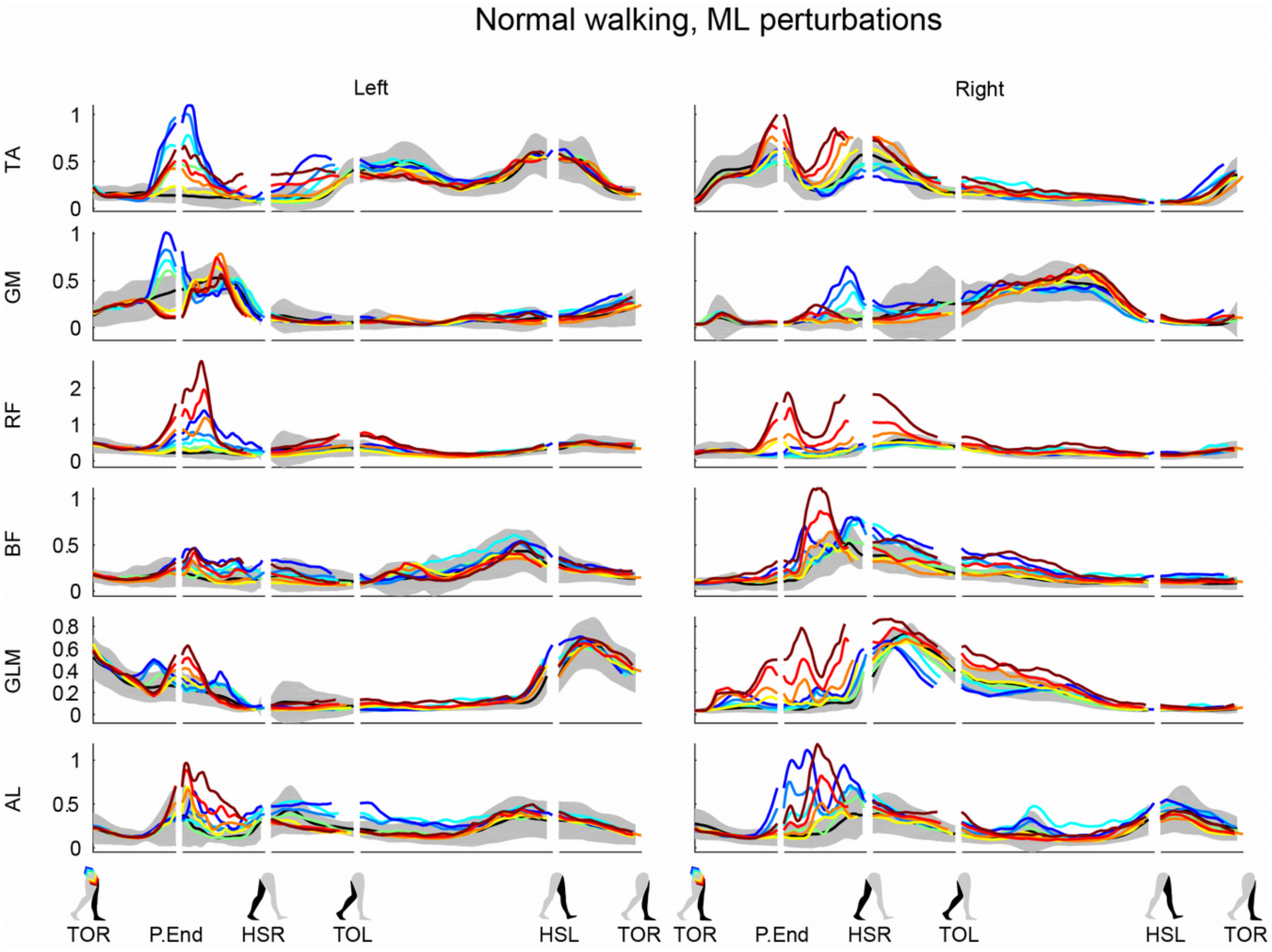

Normal walking, ML perturbations
Left
Right
TA
GM
RF
BF
GLM
AL
TOR
P.End
HSR
TOL
HSL
TOR
TOR
P.End
HSR
TOL
HSL
TOR

Fig. S22

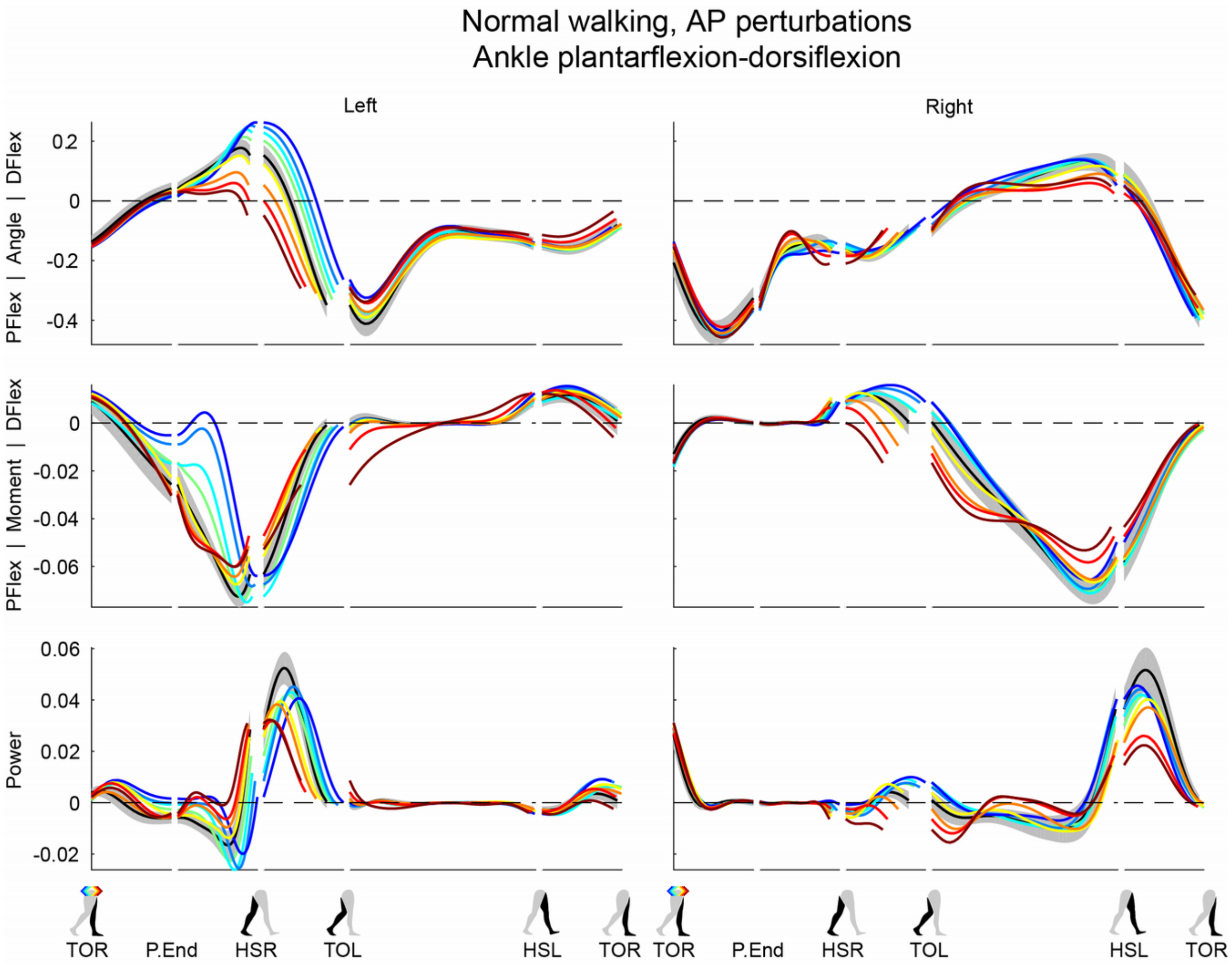

Normal walking, AP perturbations
Ankle plantarflexion-dorsiflexion
Left
Right
PFlex | Angle | DFlex
0.2
0
-0.2
-0.4
PFlex | Moment | DFlex
0
-0.02
-0.04
-0.06
Power
0.06
0.04
0.02
0
-0.02
TOR
P.End
HSR
TOL
HSL
TOR
TOR
P.End
HSR
TOL
HSL
TOR

Fig. S23

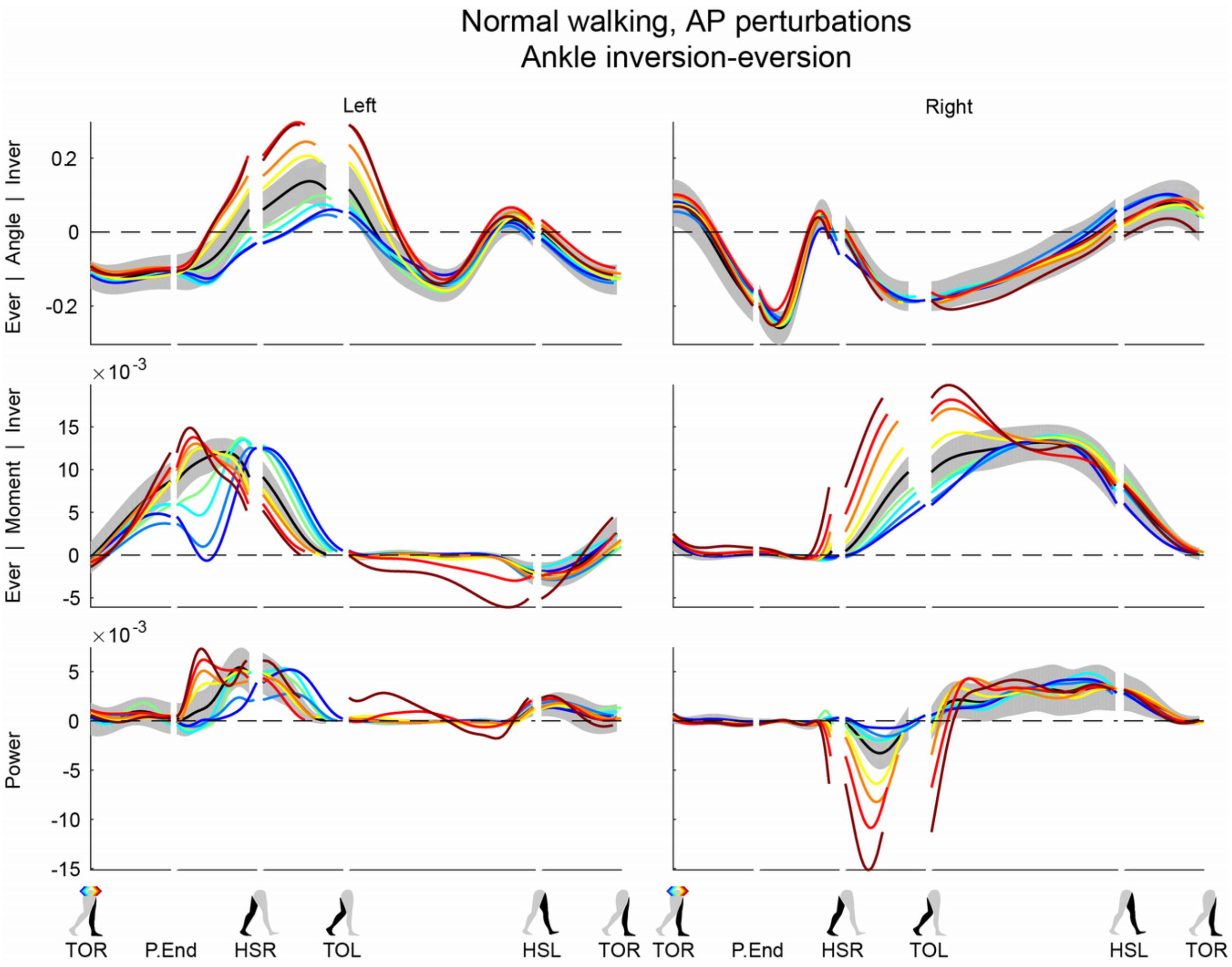

Normal walking, AP perturbations
Ankle inversion-eversion
Left
Right
Ever | Angle | Inver
0.2
0
-0.2
×10^-3
Ever | Moment | Inver
15
10
5
0
-5
×10^-3
Power
5
0
-5
-10
-15
TOR
P.End
HSR
TOL
HSL
TOR
TOR
P.End
HSR
TOL
HSL
TOR

Fig. S24

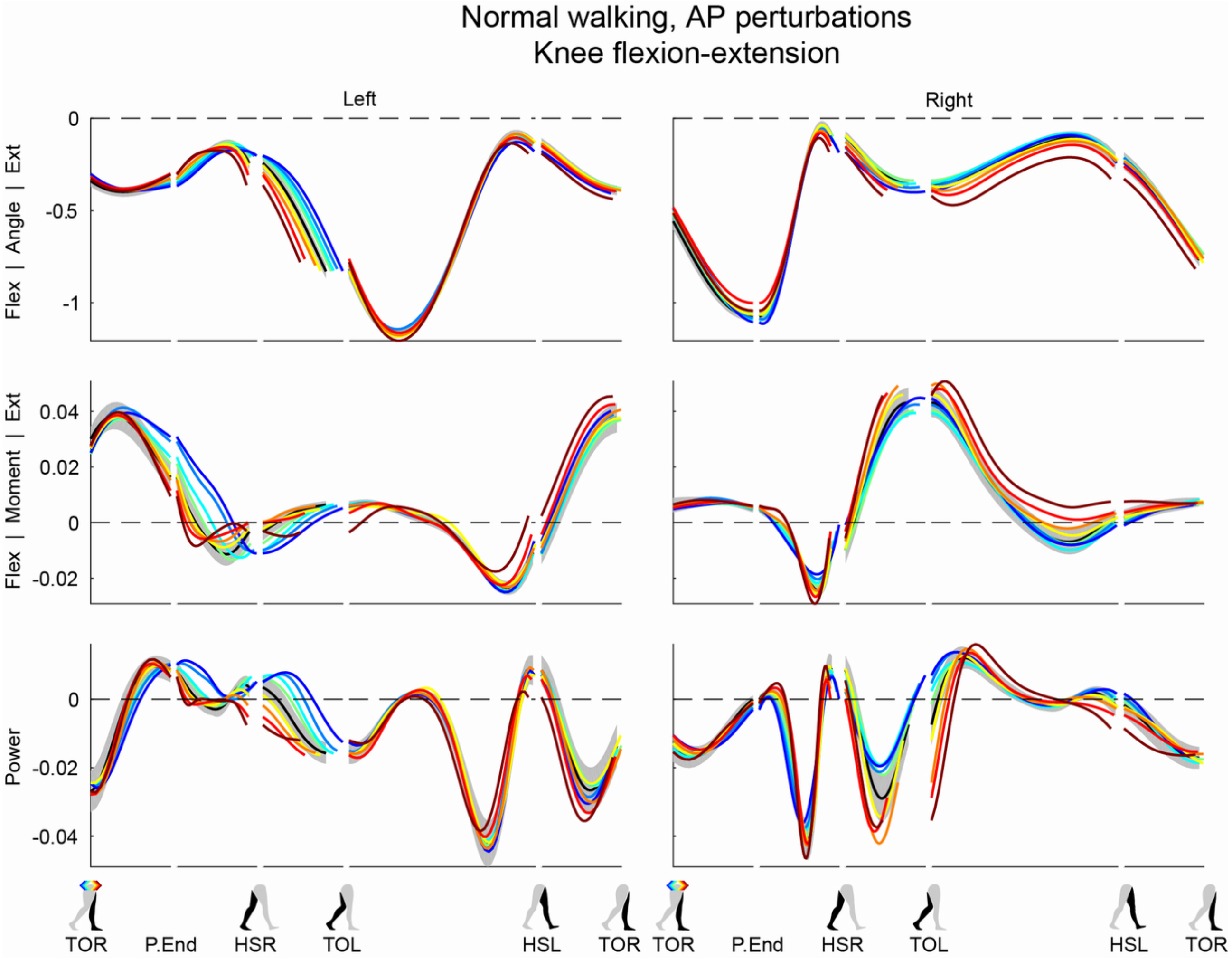

Normal walking, AP perturbations
Knee flexion-extension
Left
Right
Flex | Angle | Ext
0
-0.5
-1
Flex | Moment | Ext
0.04
0.02
0
-0.02
Power
0
-0.02
-0.04
TOR
P.End
HSR
TOL
HSL
TOR
TOR
P.End
HSR
TOL
HSL
TOR

Fig. S25

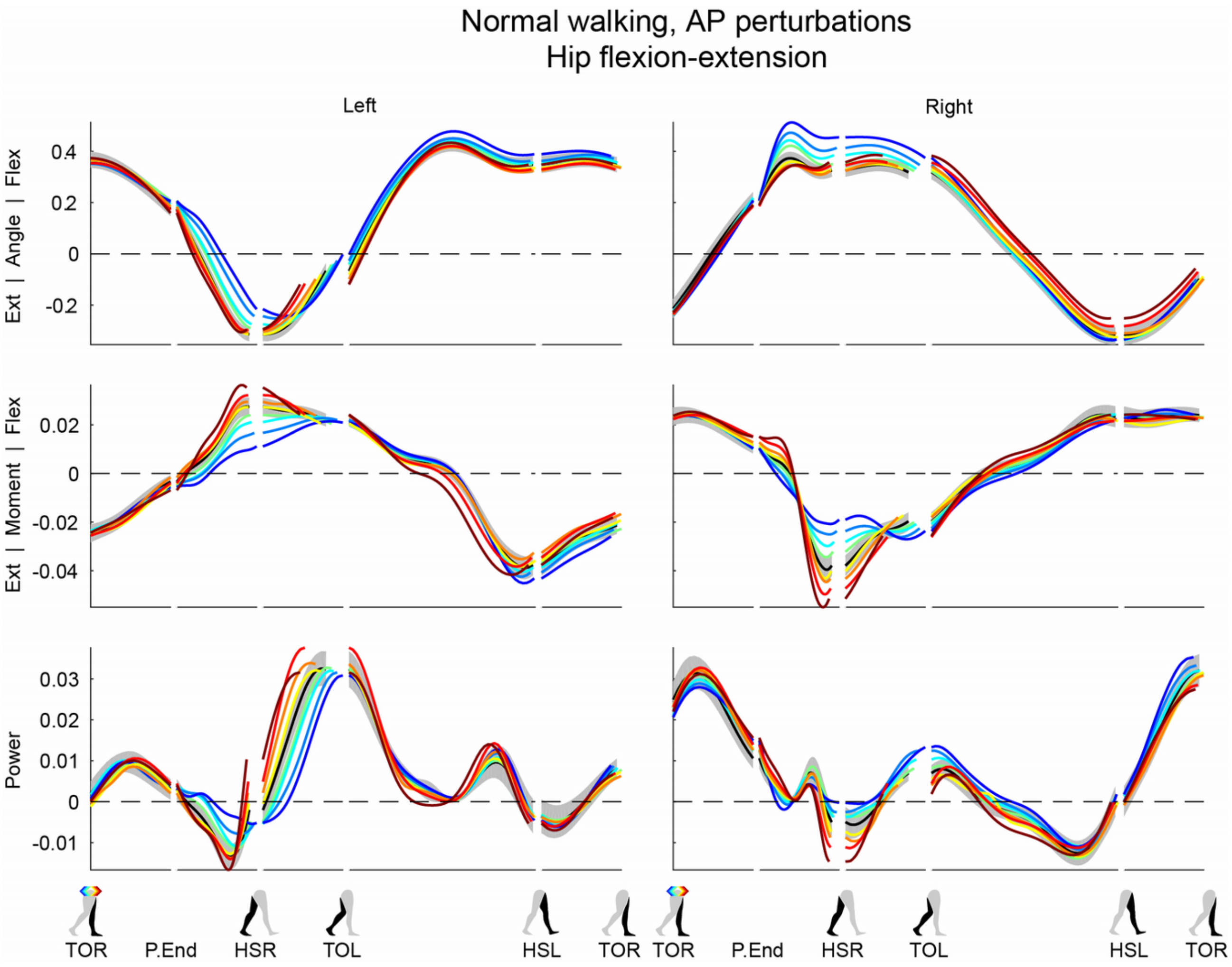

Normal walking, AP perturbations
Hip flexion-extension
Left
Right
Ext | Angle | Flex
0.4
0.2
0
-0.2
Ext | Moment | Flex
0.02
0
-0.02
-0.04
Power
0.03
0.02
0.01
0
-0.01
TOR
P.End
HSR
TOL
HSL
TOR
TOR
P.End
HSR
TOL
HSL
TOR

Fig. S26

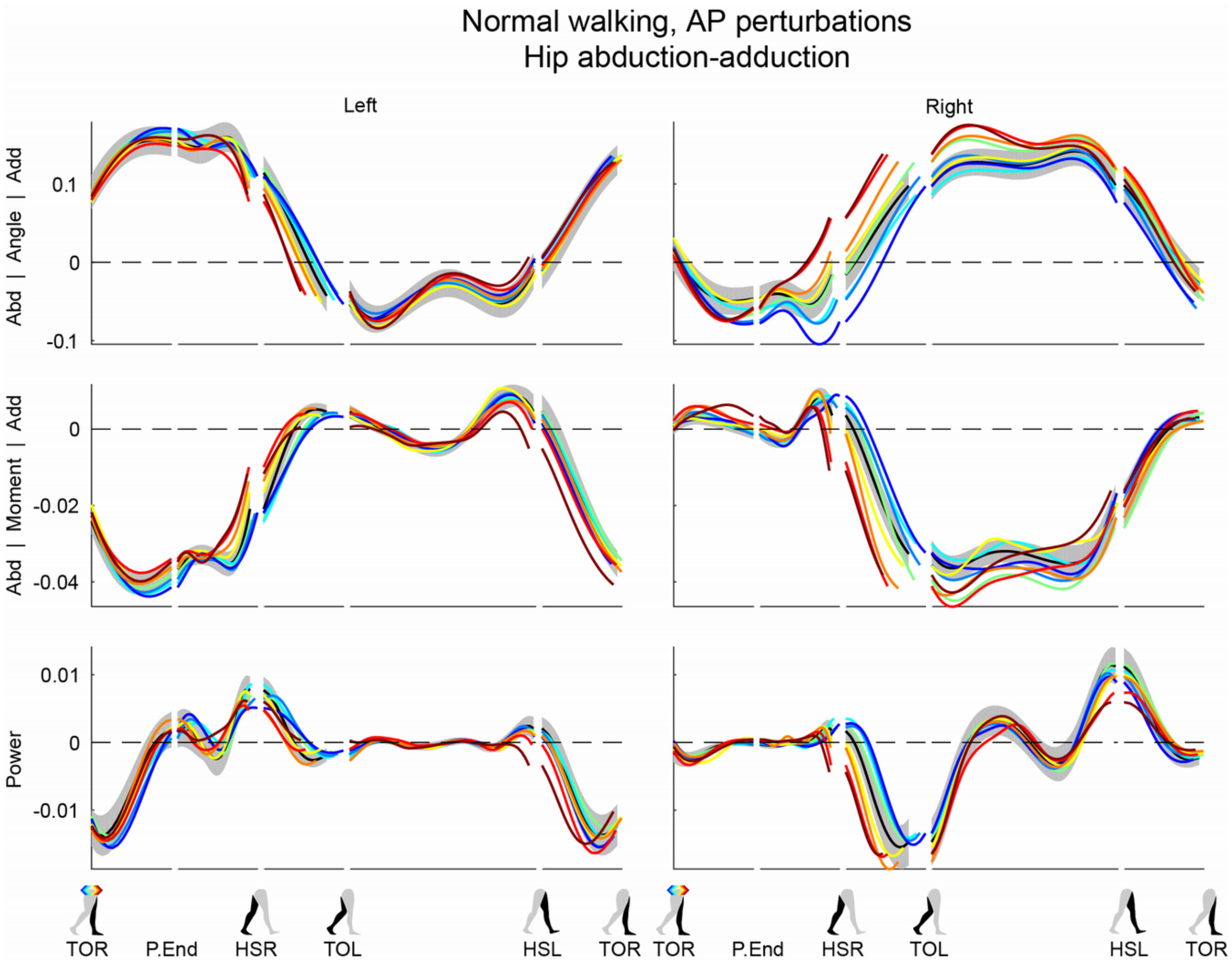
Normal walking, AP perturbations
Hip abduction-adduction
Left
Right
Abd | Angle | Add
0.1
0
-0.1
Abd | Moment | Add
0
-0.02
-0.04
Power
0.01
0
-0.01
TOR
P.End
HSR
TOL
HSL
TOR
TOR
P.End
HSR
TOL
HSL
TOR

Fig. S27

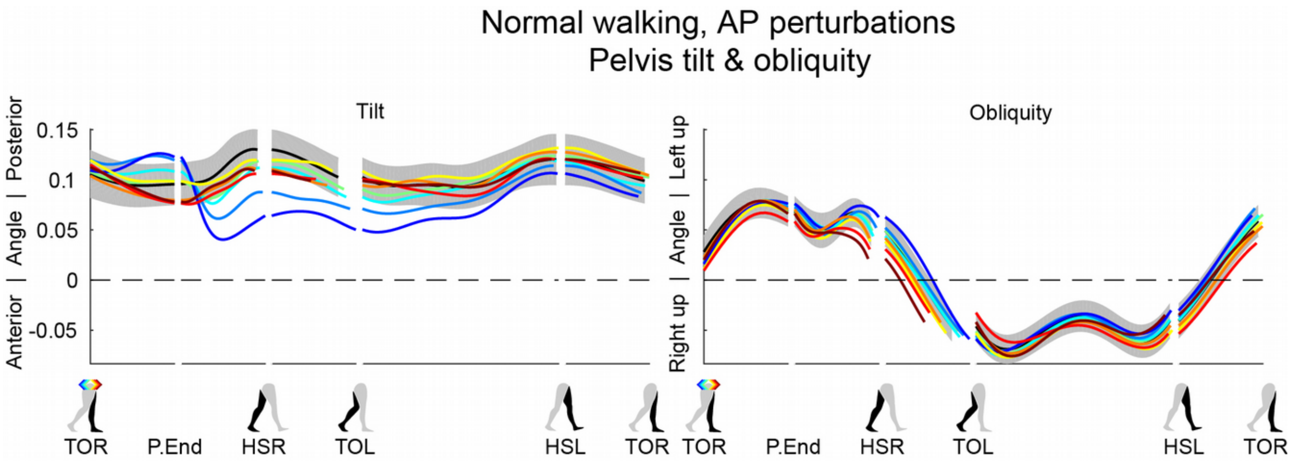

Normal walking, AP perturbations
Pelvis tilt & obliquity
Tilt
Obliquity
Anterior | Angle | Posterior
0.15
0.1
0.05
0
-0.05
Right up | Angle | Left up
TOR
P.End
HSR
TOL
HSL
TOR
TOR
P.End
HSR
TOL
HSL
TOR

Fig. S28

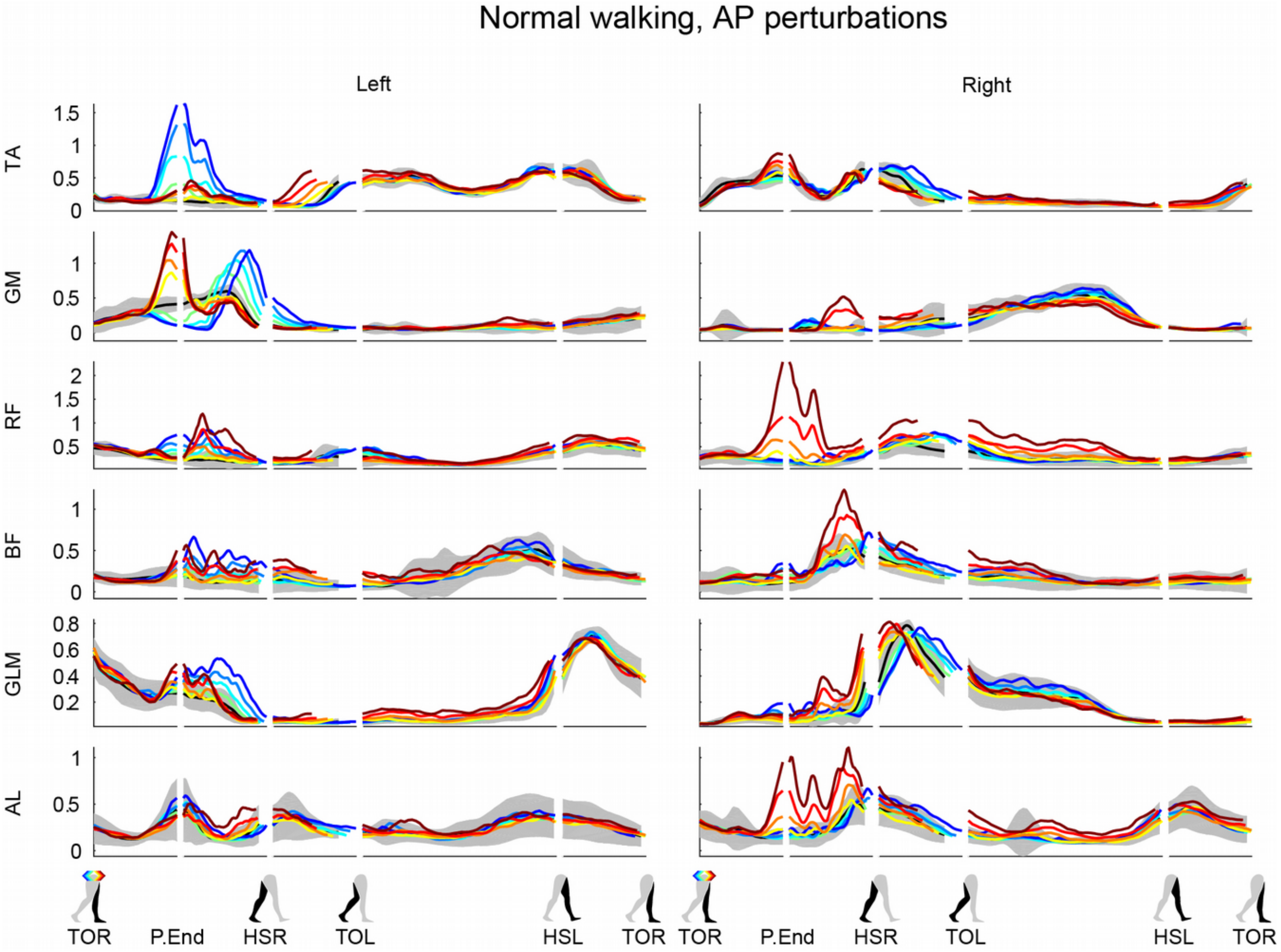

Normal walking, AP perturbations
Left
Right
TA
GM
RF
BF
GLM
AL
1.5
1
0.5
0
2
0.8
0.6
0.4
0.2
TOR
P.End
HSR
TOL
HSL
TOR
TOR
P.End
HSR
TOL
HSL
TOR
